# Expansion of Lysine-rich Repeats in *Plasmodium* Proteins Generates Novel Localization Sequences That Target the Periphery of the Host Erythrocyte*[Fn FN2]

**DOI:** 10.1074/jbc.M116.761213

**Published:** 2016-10-24

**Authors:** Heledd M. Davies, Konstantinos Thalassinos, Andrew R. Osborne

**Affiliations:** From the Institute of Structural and Molecular Biology, Department of Biological Sciences, Birkbeck and University College London, London WC1E 6BT, United Kingdom

**Keywords:** cytoskeleton, host-pathogen interaction, intracellular trafficking, intrinsically disordered protein, malaria, Plasmodium, protein evolution, protein targeting, low complexity sequences, tandem repeats

## Abstract

Repetitive low complexity sequences, mostly assumed to have no function, are common in proteins that are exported by the malaria parasite into its host erythrocyte. We identify a group of exported proteins containing short lysine-rich tandemly repeated sequences that are sufficient to localize to the erythrocyte periphery, where key virulence-related modifications to the plasma membrane and the underlying cytoskeleton are known to occur. Efficiency of targeting is dependent on repeat number, indicating that novel targeting modules could evolve by expansion of short lysine-rich sequences. Indeed, analysis of fragments of GARP from different species shows that two novel targeting sequences have arisen via the process of repeat expansion in this protein. In the protein Hyp12, the targeting function of a lysine-rich sequence is masked by a neighboring repetitive acidic sequence, further highlighting the importance of repetitive low complexity sequences. We show that sequences capable of targeting the erythrocyte periphery are present in at least nine proteins from *Plasmodium falciparum* and one from *Plasmodium knowlesi*. We find these sequences in proteins known to be involved in erythrocyte rigidification and cytoadhesion as well as in previously uncharacterized exported proteins. Together, these data suggest that expansion and contraction of lysine-rich repeats could generate targeting sequences *de novo* as well as modulate protein targeting efficiency and function in response to selective pressure.

## Introduction

Tandemly repeating protein sequences are common in most eukaryotes but are particularly abundant in protozoan parasites such as *Plasmodium falciparum* (1, 2), the species responsible for the most severe form of malaria in humans. Repetitive sequences can form through slipped strand mispairing during DNA replication or unequal crossover of chromosomes in meiosis (3). This is a dynamic process with repetitive sequences often expanding and contracting at a greater rate than that of single nucleotide mutation (4). Over half of the open reading frames in the parasite genome encode repetitive sequences (1), from modular arrays of folded domains to polyasparagine sequences, which are prone to aggregation during malarial fevers (5, 6). Hydrophobic residues are underrepresented in many *P. falciparum* repetitive sequences (7), and these are therefore predicted to be intrinsically disordered (8). To date, very few repetitive sequences of this variety have been characterized.

The host erythrocyte undergoes drastic changes during the blood stage of the parasite life cycle (9–11). Based on the presence of a conserved *Plasmodium* export element (PEXEL)^2^ or host-targeting (HT) motif, >400 proteins are predicted to be exported by *P. falciparum* into the infected cell (12, 13). These proteins, as well as a group of PEXEL-negative exported proteins (14), mediate erythrocyte modifications necessary for the parasite to survive; the nutrient-permeability of the membrane increases (15), and protrusions referred to as knobs are assembled at the erythrocyte plasma membrane. These spiral-shaped scaffolds present proteins from the PfEMP1 (*P. falciparum* erythrocyte membrane protein 1) family on the erythrocyte surface, which mediate the adhesion of infected erythrocytes to blood vessel endothelial cells (16–18). The erythrocyte cytoskeleton, which is composed of flexible α and β-spectrin filaments (19), is also rigidified upon infection (20). Cytoadhesion and the increased rigidity of infected cells contribute to parasite sequestration in specific tissues; sequestered parasites evade clearance in the spleen and are linked to severe disease outcomes, such as cerebral malaria (21).

Many proteins associated with erythrocyte rigidification and cytoadhesion contain tandem repeats (22–29), yet their role in protein function remains unclear. Some repeating sequences appear to be under immune selection (30), and many are highly antigenic (31); it has been proposed that this may allow the parasite to evade the host immune system by diverting B-cell responses toward non-protective epitopes (32) or promoting an inferior T-cell-independent maturation of B-cells (33, 34). Such general roles for tandemly repeating sequences may explain their broad distribution in parasite proteins.

Repetitive sequences in some proteins may be removed with no consequence for protein function (35), suggesting that they are encoded by functionally neutral “junk DNA” that has expanded due to errors in DNA replication. However, removal of the repetitive regions of other proteins can affect activity; deletion of repeat regions of the parasite circumsporozoite protein and ring-exported protein 1 (REX1) lead to loss of protein function (36, 37). The knob-associated histidine-rich protein (KAHRP) is involved in both rigidifying the host cell (23) and the formation of cytoadherent knob structures (16), and deletion of a C-terminal sequence encompassing two lysine-rich repetitive sequences results in smaller knob structures and reduced cytoadhesion (38). The Lysine-rich membrane-associated PHISTb protein (LYMP) also modulates cytoadhesion (39). PHISTb proteins are a subgroup of the PHIST family of exported proteins that contain a *Plasmodium* RESA N-terminal (PRESAN) domain (40, 41). Several PRESAN domain-containing proteins have been shown to localize to the erythrocyte periphery (42, 43), and in the case of LYMP, this domain has been shown to bind to PfEMP1 (44, 45). Its C terminus, which includes tandem repeats rich in lysine, has been shown to interact with the cytoskeletal component band 3 (44). The role of tandemly repeating sequences in functionally important regions of both LYMP and KAHRP suggests that these are not erroneous expansions but may be directly involved in modulating the cytoadhesive properties of the infected host cell.

Other known cytoskeleton-binding proteins also contain repetitive sequences, many of which are rich in lysine and glutamate residues (46, 47). A role for these highly charged sequences in protein function has yet to be demonstrated, and cytoskeleton-binding sites for the proteins RESA, Pf332 (*P. falciparum* protein 332), PfEMP3, MESA, and the PHISTa protein PF3D7_0402000 have previously been identified in non-repetitive regions (27, 48–53).

Here we show that lysine-rich repeating sequences constitute targeting modules that direct a number of exported parasite proteins to the periphery of the infected erythrocyte. Based on the observation that targeting efficiency is dependent upon repeat length, we present a model in which repeat expansion and contraction can generate novel targeting modules or modulate the targeting efficiency of exported parasite proteins.

## Results

### 

#### 

##### Multiple Lysine-rich Repeating Sequences within Glutamic Acid-rich Protein (GARP) Localize to the Infected Erythrocyte Periphery

**FIGURE 1.**
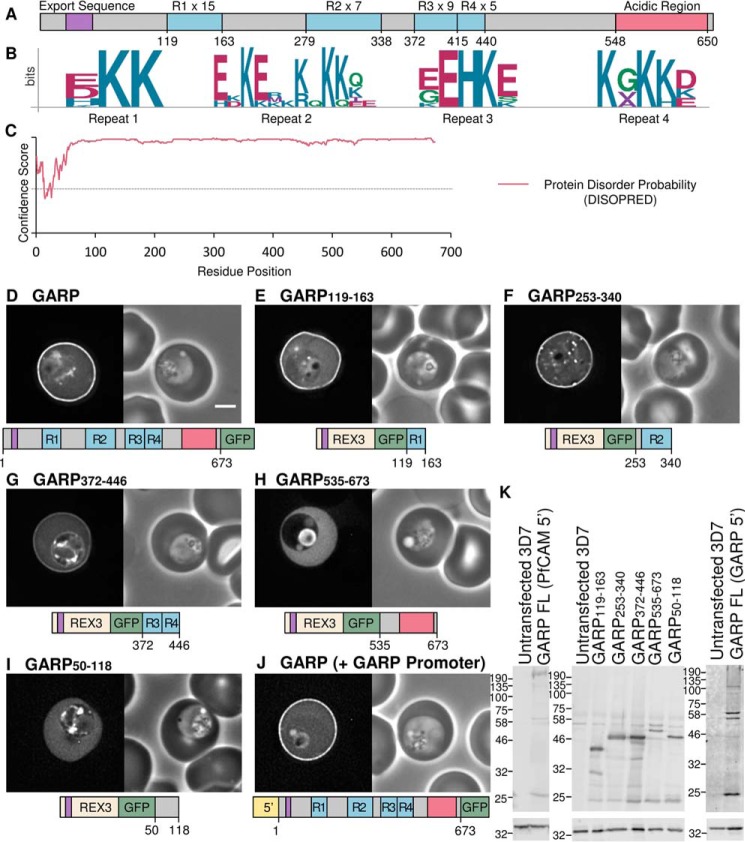
**GARP is targeted to the erythrocyte periphery by three lysine-rich repeat regions.** *A*, representation of the GARP protein. The lysine-rich repeating regions are *highlighted* in *blue* and *labeled R1–R4*, and the number of repeating units in each is indicated. The acidic C terminus is shown in *red*. The export sequence is *colored purple* and represents both the signal sequence and PEXEL/HT motif. *B*, sequence logos for the four lysine-rich repeats; residue position is shown on the *x* axis, and conservation is indicated on the *y* axis (*bits*). *C*, disorder prediction for GARP (using DISOPRED (55)). Amino acids are considered disordered if they have a confidence score >0.5, represented by a *dotted line. D–I*, GFP-tagged full-length GARP and truncations expressed using the calmodulin promoter in *P. falciparum* parasites. *J*, GFP-tagged full-length GARP expressed using the GARP promoter. GFP fluorescence and phase-contrast images are shown on the *left* and *right*, respectively. A representation of each construct is shown *below. Scale bar*, 2 μm. For quantification of fluorescence, see supplemental Fig. 1 and Table 2. *K*, anti-GFP Western blot (*top*), with anti-HAP used to confirm equal loading (*bottom*).

GARP is an 80-kDa protein encoded by the *P. falciparum* gene PF3D7_0113000 (54). It contains an N-terminal signal sequence for targeting to the parasite endoplasmic reticulum and a PEXEL/HT motif sequence, RLLNE, enabling the protein to be exported into the host erythrocyte. GARP is a highly charged protein; it contains 24% glutamic acid, 21% lysine, and 9% aspartic acid residues. These charged residues are concentrated within six tandemly repeated sequences, which each contain a unique repeated motif. The first four repeat sequences are lysine-rich, and the C terminus of the protein contains an acidic stretch composed of two different repeating units (Fig. 1, *A* and *B*, and Table 1). Beyond the N-terminal signal sequence, GARP contains very few hydrophobic residues, suggesting that it does not contain stable folded domains. Indeed, protein disorder analysis using the program DISOPRED (55) suggests that the entire sequence of GARP is intrinsically disordered (Fig. 1*C*).

**TABLE 1:**
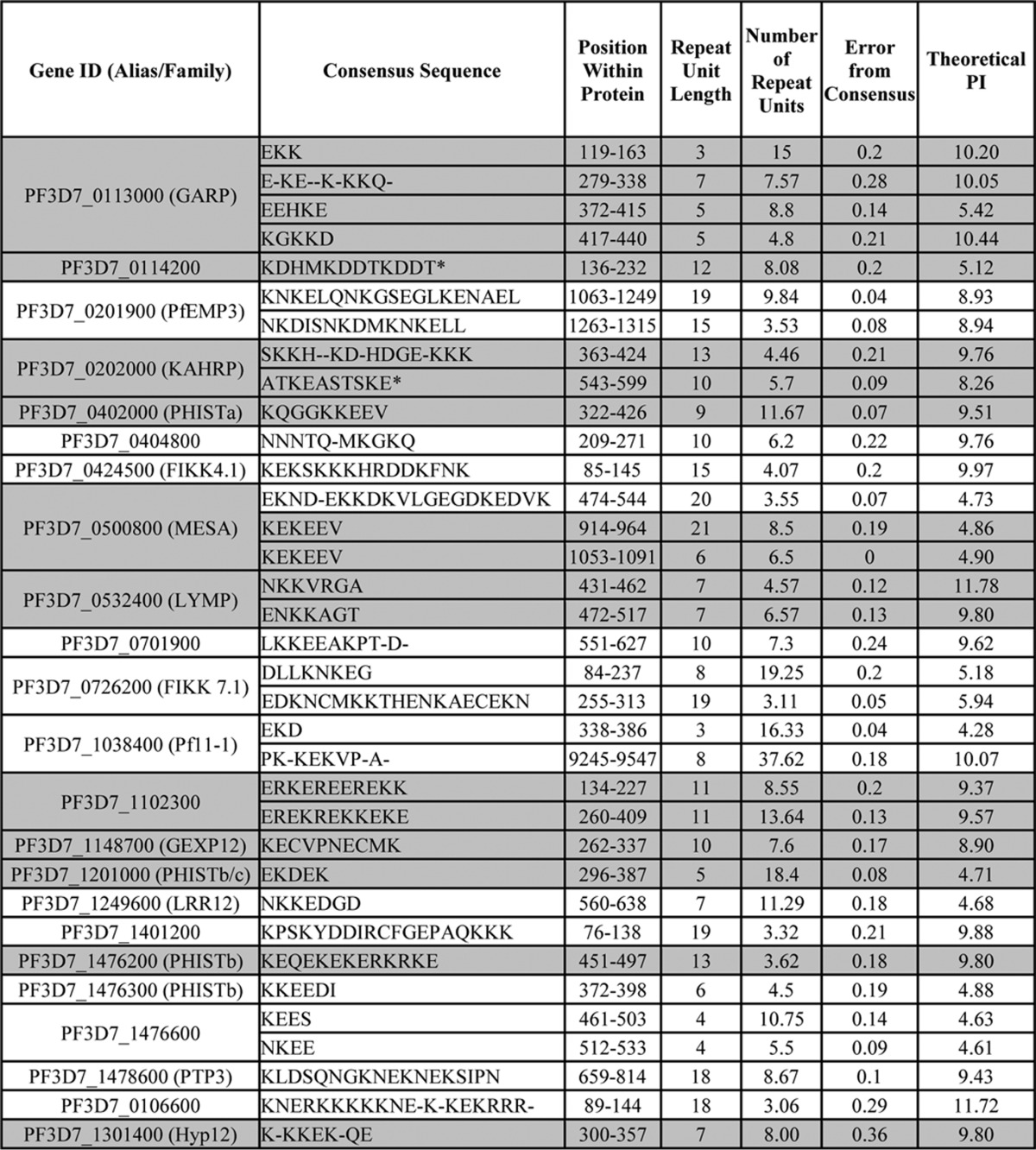
***P. falciparum* proteins with charged repeat sequences predicted to target to the erythrocyte periphery** Proteins selected to be GFP-tagged and expressed in *P. falciparum* are highlighted in grey. Asterisks indicate tested sequences which did not localise to the erythrocyte periphery. The consensus sequence, position within the protein, repeat unit length, number of repeat units, and the error from consensus were defined by XSTREAM (110). Non-integer numbers of repeat units indicate degeneration at the ends of repetitive sequences. Theoretical pI calculated by PROTPARAM is shown for each fragment (112).

To determine the localization of GARP, the protein was GFP-tagged and expressed in the blood stage of *P. falciparum* parasites using the calmodulin promoter. GFP fluorescence was localized at the periphery of the red blood cell, indicating that the protein is recruited either to the plasma membrane or the adjacent spectrin cytoskeleton of the infected erythrocyte (Fig. 1*D*). Quantification of relative fluorescence intensity indicated a 3.27 ± 0.86-fold increase in fluorescence intensity at the erythrocyte periphery relative to the cytoplasm (see supplemental Fig. 1 and Table 2 for additional images and quantification of all parasite lines).

**TABLE 2:** **Quantification and Statistical Analysis of GFP Fluorescence at the Periphery of Infected Erythrocytes** The -fold difference in fluorescence intensity at the erythrocyte membrane relative to the cytosol was calculated as described in the legend to supplemental Fig. 1. Statistical analysis using one-way analysis of variance was performed, and multiple comparisons were made between each parasite line and a line expressing GFP-tagged REX3_1–60_ only. Images of 20 parasites were quantified per parasite line (*n* = 20). *p* values and levels of significance are indicated, from not significant (ns) to extremely significant (*** and ****). FL, full-length.

Construct	Difference in fluorescence at membrane relative to cytosol	S.D.	*p* (significance)
	*-fold*		
REX3(1–61)	0.92	0.06	
Full-length GARP	3.27	0.86	<0.0001 (****)
GARP(119–163)	2.39	0.43	<0.0001 (****)
GARP(253–340)	3.23	0.61	<0.0001 (****)
GARP(372–446)	1.71	0.29	<0.0001 (****)
GARP(535–673)	0.92	0.07	0.9988 (ns)
GARP(50–118)	0.92	0.06	0.9548 (ns)
GARP (+promoter)	2.90	0.72	<0.0001 (****)
GARP(119–163) (+linker)	2.29	0.60	<0.0001 (****)
GARP(134–163)	1.76	0.29	<0.0001 (****)
GARP(149–163)	1.12	0.20	0.2323 (ns)
GARP(372–446) (+linker)	1.76	0.37	<0.0001 (****)
*P. reichenowi* GARP(71–130)	0.90	0.06	0.9181 (ns)
*P. gaboni* GARP(381–412)	0.94	0.07	0.9248 (ns)
PF3D7_1102300(121–415)	3.90	0.65	<0.0001 (****)
GEXP12(231–370)	1.75	0.35	<0.0001 (****)
LYMP(419–528)	2.13	0.34	<0.0001 (****)
PF3D7_1476200(443–512)	2.50	0.57	<0.0001 (****)
PF3D7_0402000(305–428)	1.73	0.40	<0.0001 (****)
PF3D7_1201000(292–397)	1.42	0.45	0.0016 (**)
MESA(850–1147)	1.38	0.20	0.0062 (**)
KAHRP(363–428)	1.49	0.21	0.0007 (***)
KAHRP(540–600)	0.91	0.08	0.9528 (ns)
PF3D7_0114200(97–240)	0.92	0.04	0.9845 (ns)
PF3D7_1149100.1(120–416)	0.92	0.05	0.9917 (ns)
Hyp12(297–381)	2.31	0.33	<0.0001 (****)
PKNH_1325700(303–445)	3.12	0.67	<0.0001 (****)
PF3D7_1102300 (FL)	4.08	1.06	<0.0001 (****)
GEXP12 (FL)	1.97	0.44	<0.0001 (****)
PF3D7_0402000 (FL)	3.73	0.80	<0.0001 (****)
PF3D7_1201000 (FL)	1.09	0.20	0.2920 (ns)
Hyp12 (FL)	0.94	0.08	0.9064 (ns)
Hyp12(51–381)	1.00	0.10	1.82403 (ns)
Hyp12(158–381)	0.65	2.35	<0.0001 (****)
PF3D7_1102300 (+promoter)	4.01	1.82	<0.0001 (****)
PF3D7_1476200 (+promoter)	2.35	0.52	<0.0001 (****)

Because GARP is composed mainly of repetitive, low complexity, and intrinsically disordered sequences, it is likely that at least some of these sequences constitute novel modules that can target a protein to the periphery of the infected cell. To test this, fragments encoding the three lysine-rich repeating sequences were GFP-tagged and fused to the N-terminal signal sequence and PEXEL/HT motif of the protein REX3 (residues 1–61), which has been used previously to mediate protein export (Fig. 1, *E–G*) (41, 43). This REX3 fragment alone does not target proteins to the erythrocyte periphery (supplemental Fig. 1*B*). The first lysine-rich repeat sequence contains a three-residue motif that is repeated 15 times. The consensus sequence of the repeated motif, defined by the program XSTREAM, is EKK. The first residue in this motif varies (represented by E, D, H, or K residues), but the two lysine residues are highly conserved (Fig. 1*B*). A GFP fusion protein containing the first lysine-rich repeat region (GARP(119–163)) is efficiently exported and localized to the periphery of the infected erythrocyte (Fig. 1*E*). GARP(253–340), which contains the second lysine-rich repeat comprising seven repeats of the degenerate amino acid sequence E-KE-K-KKQ- (where a hyphen indicates that a gap is most commonly found at a particular position), is also efficiently localized to the erythrocyte periphery (Fig. 1, *B* and *F*). Similarly, GARP(372–446), encompassing the third and fourth repeats, which are immediately adjacent and comprise nine repeats of the sequence EEHKE followed by five repeats of the sequence KGKKD, also exhibits a clear localization at the periphery of the infected erythrocyte (Fig. 1, *B* and *G*). Conversely, the acidic C terminus of GARP, GARP(535–673), remains in the erythrocyte cytosol (Fig. 1*H*). GFP accumulation in the food vacuole is also seen in some parasites, probably due to endocytosis of the erythrocyte cytoplasm by the parasite. This is also seen in other parasite lines but is generally less apparent when proteins are localized to the erythrocyte periphery because this probably reduces the efficiency with which these proteins are endocytosed. Likewise, the uncharged N terminus of the protein, GARP(50–118), is not peripherally targeted (Fig. 1*I*). Expression of all proteins was confirmed by Western blotting (Fig. 1*K*). The full-length GARP protein appears as a blurred band, and most constructs migrate at a mass higher than that expected; this is probably due to the highly charged and repeating nature of the proteins. Taken together, these data indicate that at least three lysine-rich repeating and intrinsically disordered regions within GARP are sufficient to form targeting modules that localize to the erythrocyte periphery.

##### The Targeting Efficiency of Lysine-rich Repeat Sequences Is Length-dependent

**FIGURE 2.**
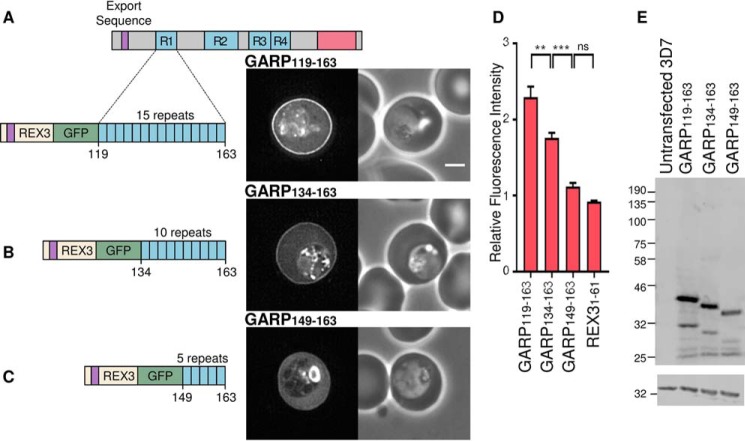
**Truncating the first charged repeat of GARP decreases targeting efficiency.** *A–C*, truncated fragments of the first lysine-rich repeat sequence (*blue*) of GARP, GFP-tagged and expressed in *P. falciparum* parasites. Expressed proteins are shown on the *left*, with *dotted lines* indicating the region cloned from the full-length protein. The export sequence (*purple*) of REX3 was used to drive export of each fragment. GFP fluorescence and phase-contrast images are shown on the *left* and *right*, respectively. *Scale bar*, 2 μm. *D*, the ratio of the fluorescence intensity adjacent to the erythrocyte membrane relative to the erythrocyte cytoplasm for the indicated proteins is shown. *Error bars*, S.E. *ns*, *, **, ***, and ****, not significant (*p* > 0.05), *p* ≤ 0.05, *p* ≤ 0.01, *p* ≤ 0.001, and *p* ≤ 0.0001, respectively. *E*, anti-GFP Western blot (*top*), with anti-HAP used to confirm equal loading (*bottom*).

Because each periphery-targeting sequence of GARP is repetitive in character, we tested whether the length of the lysine-rich sequence affects its targeting efficiency. The first lysine-rich repeat sequence of GARP was truncated from 45 residues to 30 and 15 residues, containing 15, 10, and 5 repeats, respectively (Fig. 2). An additional linker sequence of 12 residues was inserted between GFP and the GARP fragments to ensure that proximity to GFP did not compromise potential interactions of the lysine-rich fragments. As expected, GARP(119–163), which encodes all 15 repeats, is localized at the erythrocyte periphery, indicating that the addition of the linker sequence does not alter the targeting function of the first lysine-rich repeat sequence (Fig. 2, *A* and *D*). GARP(134–163), which contains only 10 repeats, is also localized to the erythrocyte periphery, but targeting is less efficient (Fig. 2, *B* and *D*); fluorescence intensity at the erythrocyte periphery relative to the erythrocyte cytoplasm is reduced. The shortest construct, GARP(149–163), only encodes five repeats and is not efficiently recruited to the periphery; the protein is predominantly localized diffusely in the erythrocyte cytoplasm (Fig. 2, *C* and *D*). Expression of each of the proteins was confirmed by Western blotting (Fig. 2*E*).

These data show that multiple repeats are required for the targeting of lysine-rich sequences to the erythrocyte periphery and that the efficiency of targeting increases as the number of repeats increases. In the context of the first GARP repeat, a sequence of ∼30 amino acids in length is necessary for robust peripheral targeting.

##### Expansion of Repeating Lysine-rich Sequences Can Generate Sequences with a Targeting Function in Exported Parasite Proteins

Repetitive DNA sequences are highly mutable and are prone to expansion and contraction (4). Given the preceding data, this suggests that sequences with a peripheral targeting function may arise *de novo* simply by expansion of short non-functional lysine-rich motifs.

**FIGURE 3.**
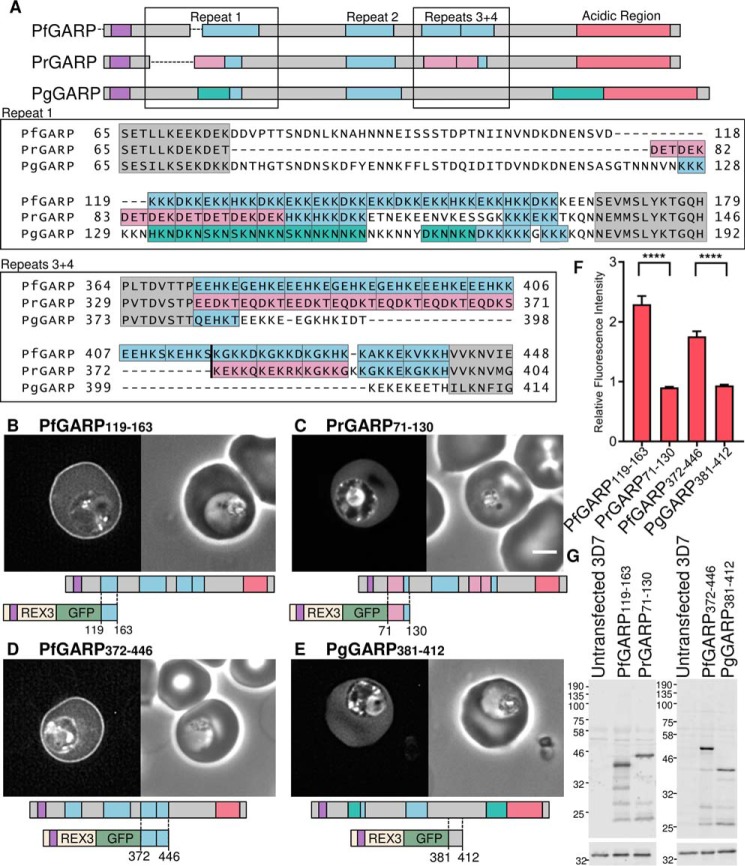
**Repeat expansion and generation of functional targeting sequences in parasite proteins.** *A*, representation of *P. falciparum* GARP (*top*), *P. reichenowi* GARP (*middle*), and *P. gaboni* GARP (*bottom*) with the export sequence in *purple* and the acidic C terminus in *red*. Lysine-rich repeats are shown in *blue*. Repeated sequences corresponding to the *P. falciparum* first lysine-rich sequence motif (E/K/D/H)KK are also shown in *blue*, and the alternative expanded repeat motifs DE(T/K) from *P. reichenowi* and (H/D/N)KN from *P. gaboni* are shown in *pink* and *turquoise*, respectively. Alignment of lysine-rich repeat 1 and lysine-rich repeats 3 and 4 of PfGARP and the homologous regions in PrGARP and PgGARP are shown *below. P. falciparum* lysine-rich repeats are *boxed* in *blue*, *P. reichenowi* motifs in *pink*, and *P. gaboni* motifs in *turquoise*. Flanking conserved sequences are *shaded gray. B* and *C*, GFP-tagged PfGARP (*B*) and PrGARP regions (*C*) encompassing the first repeat expressed in *P. falciparum. D* and *E*, GFP-tagged PfGARP (*D*) and PrGARP regions (*E*) encompassing both the third and fourth repeats expressed in *P. falciparum*. A GFP fluorescence image and a phase-contrast image are shown on the *left* and *right*, respectively. A *schematic* of the cloned region is shown *below. Scale bar*, 2 μm. *F*, the ratio of the fluorescence intensity at the erythrocyte periphery relative to the erythrocyte cytoplasm for the indicated proteins is shown, as in Fig. 2. *G*, anti-GFP Western blot of parasites (*top*). Anti-HAP was used to confirm equal loading (*bottom*). *Error bars*, S.E.

To test whether this phenomenon can be observed over evolutionary time, we compared the GARP sequences of *P. falciparum* with those of closely related *Plasmodium* species (56, 57). The *P. falciparum* and *Plasmodium reichenowi* genes encoding GARP are syntenic; the latter also encodes an exported protein that contains four lysine-rich repeats and a C-terminal acidic sequence. Whereas the first lysine-rich repeat of the *P. falciparum* protein corresponds to 15 copies of the (E/D/K/H)KK motif, the first lysine-rich repeat of PrGARP contains only five repeats conforming to this consensus (Fig. 3*A*). Instead, in the *P. reichenowi* protein, a more acidic DE(T/K) repeat has expanded in this region (Fig. 3*A*). Analysis of the equivalent *Plasmodium gaboni* GARP sequence indicates that yet another repeat motif, (H/D/N)KN, has expanded in addition to four repeats of the (E/D/K/H)KK motif (57).

Although the second GARP repeat sequence is similar in all three parasite species, the third and fourth sequences in the *P. gaboni* protein have not expanded and comprise only one or two highly degenerate repeats (Fig. 3*A*).

To test whether the expansion of the first, third, and fourth repeat sequences has led to the formation of functional targeting sequences in *P. falciparum* GARP, the localization of GFP fusion proteins derived from these sequences from different species was compared. Consistent with this model, the GFP-tagged first repeat from *P. falciparum* GARP (PfGARP(119–163)) is localized to the erythrocyte periphery (Fig. 3, *B* and *F*), but the equivalent GFP-tagged *P. reichenowi* GARP fragment (PfGARP(71–130)), which contains fewer lysine-rich repeats, is diffusely localized in the erythrocyte cytoplasm (Fig. 3, *C* and *F*). Likewise, the region of PfGARP (PfGARP(372–446)), comprising the third and fourth repeats, is localized to the red cell periphery (Fig. 3, *D* and *F*), but the equivalent region from the *P. gaboni* protein (PgGARP(381–412)) is not (Fig. 3, *E* and *F*). Anti-GFP Western blotting confirmed the expression of proteins at the expected size (Fig. 3*G*).

Although the sequence of the common ancestor of these proteins is not known, these experiments suggest that expansion of non-functional, short lysine-rich repeats can lead to the formation of novel protein modules that can direct the localization of exported parasite proteins within the infected erythrocyte.

##### Lysine-rich Repeat Regions from Multiple Exported P. falciparum Proteins Confer Peripheral Localization in the Infected Erythrocyte

Many *Plasmodium* proteins contain repetitive sequences enriched in charged residues. To investigate whether sequences similar to those in GARP are capable of targeting to the erythrocyte periphery, we identified putative exported proteins, characterized by an N-terminal signal sequence or transmembrane domain and an R*X*L motif, that also contain repeating sequences ≥30 residues in length with a lysine content ≥20%.

Lysine-rich and repetitive sequences were identified in exported protein sequences using a sliding window algorithm and the program XSTREAM, respectively. Thirty-five sequences, including those within GARP, were found to conform to the above criteria, with some proteins containing multiple repeating lysine-rich sequences (Table 1).

**FIGURE 4.**
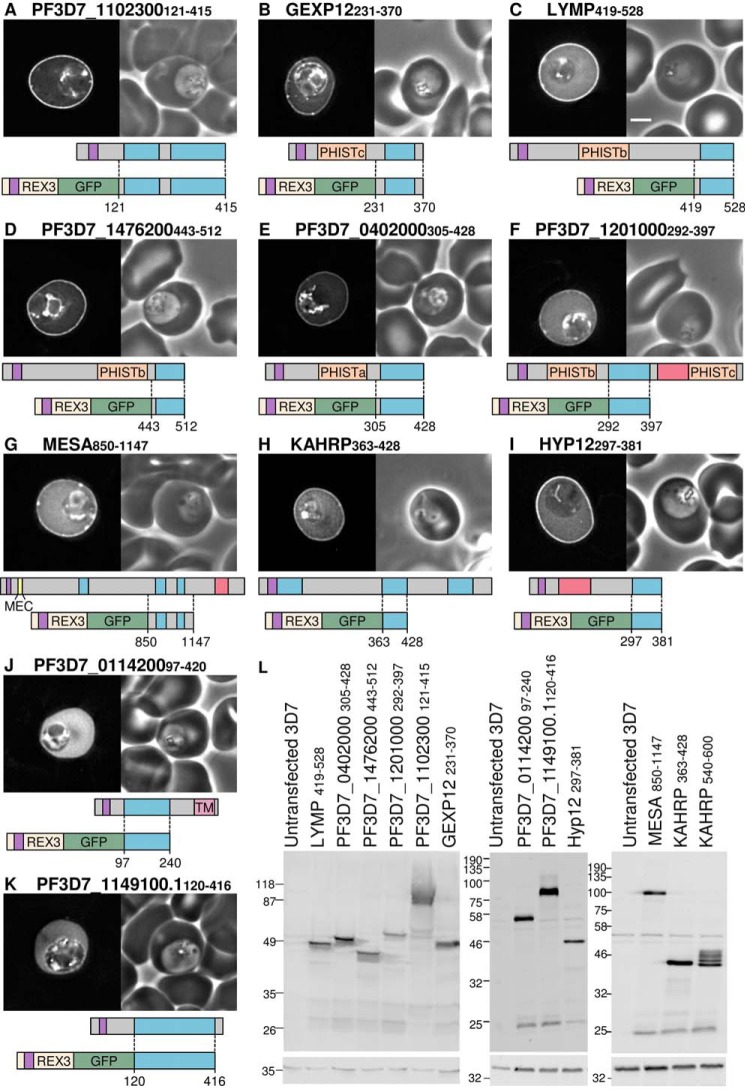
**Multiple lysine-rich repeating sequences from *P. falciparum* proteins target the erythrocyte periphery.** *A–K*, identities of proteins are shown *above* each *image*. Shown are GFP localization (*left*) and a phase-contrast image (*right*). A representation of the full-length protein is shown *below* each image, with the lysine-rich repeat regions in *blue*, export sequences (signal sequence and PEXEL/HT motif) in *purple*, acidic sequences in *red*, PRESAN/PHIST domains in *orange*, predicted transmembrane domains in *pink*, and the MESA erythrocyte cytoskeleton-binding (*MEC*) motif in *yellow. Dotted lines*, protein sequence cloned into each GFP-tagged construct, shown *below* the full-length protein. Protein schematics are approximately to scale, with MESA downscaled by one-half. *Scale bar*, 2 μm. *L*, anti-GFP Western blots of all parasite lines (*top*). Anti-HAP was used to confirm equal loading (*bottom*).

Sequences encoding lysine-rich repeat sequences from 10 proteins (Table 1, highlighted) were expressed as GFP fusion proteins, and their localization was assessed by fluorescence microscopy. PF3D7_1102300 protein, like GARP, is predicted to be entirely intrinsically disordered; the majority of the sequence is lysine-rich and repeating (Fig. 4*A*). A fusion protein that included the N terminus of REX3, GFP, and the lysine-rich sequence of PF3D7_1102300 comprising residues 121–415 (PF3D7_1102300 (121–415)), was expressed in parasites. Within the infected erythrocyte, GFP fluorescence was localized at the periphery of the infected cell (Fig. 4*A*). GEXP12 (gametocyte-exported protein 12) contains an N-terminal PRESAN domain belonging to the PHISTc family and a C-terminal lysine-rich sequence. A similar pattern of peripheral GFP fluorescence is seen in erythrocytes infected with parasites expressing an exported GFP protein that includes this fragment (GEXP12(231–370)) (Fig. 4*B*); some brighter foci of fluorescence are also seen in some cells.

A number of proteins known to target the erythrocyte cytoskeleton via defined motifs in non-repeating sequences also contain lysine-rich repeating sequences that have not previously been shown to function as independent targeting domains *in vivo*. The lysine-rich repeat regions of the PHISTb proteins LYMP (LYMP(419–528)) and PF3D7_1476200 (PF3D7_1476200(443–512)) and the PHISTa protein PF3D7_0402000 (PF3D7_0402000(305–428)) were expressed as GFP fusion proteins; peripheral GFP fluorescence was seen in erythrocytes infected with all three parasite lines (Fig. 4, *C–E*, respectively). A GFP fusion protein encompassing the lysine-rich region of the PHISTb/c protein PF3D7_1201000 (PF3D7_1201000(292–397)) exhibited a weak localization at the periphery that was visible in only a fraction (50–80%) of infected cells (Fig. 4*F*).

The N terminus of MESA contains a 20-residue cytoskeleton-binding MEC motif (51). The remainder of the MESA sequence consists of various charged repetitive sequences, three of which have a lysine content of >20%. The second lysine-rich repeat sequence and flanking sequence has duplicated to form the third repeat. A GFP fusion protein that contains both of these sequences (MESA(850–1147)) also localizes to the erythrocyte periphery (Fig. 4*G*). Similarly, KAHRP contains an N-terminal histidine-rich sequence that is sufficient to target to the erythrocyte periphery (58) but also contains two lysine-rich repeat regions that are important for protein function (38). A GFP fusion protein encompassing the first of the lysine-rich repeats (5′ repeats) is also targeted to the periphery of the infected erythrocyte (Fig. 4*H*).

Hyp12 contains a lysine-rich C-terminal sequence; the repeats in the sequence are highly degenerate. When fused to GFP in the absence of other sequences, the repeat sequence localizes to the erythrocyte periphery (Fig. 4*I*).

Protein PF3D7_0114200 is predicted to contain a C-terminal transmembrane domain as well as a lysine-rich sequence. The lysine-rich sequence was fused to REX3:GFP and expressed in parasites (PF3D7_0114200(97–420)). In this case, the fluorescence remained localized within the cytosol of the erythrocyte, with no peripheral targeting (Fig. 4*J*). PF3D7_1149100.1 contains six repetitions of a 40-residue motif, but the lysine content of this sequence is only 17% lysine, and this fragment also remained in the erythrocyte cytosol (Fig. 4*K*). Additionally, the C-terminal lysine-rich repeat sequence (3′ repeats) from KAHRP (KAHRP(540–600)) does not localize to the cell periphery despite having a lysine content of 20% (supplemental Fig. 1*W*). Although it is difficult to interpret a negative result, this suggests that a certain threshold of lysine residues is required for peripheral localization within the erythrocyte and that the distribution of residues within repeats may also be important. In the case of the KAHRP 3′ repeats, the lack of peripheral targeting could also be due to partial degradation of the protein because several bands are seen on Western blots of parasites expressing this protein (Fig. 4*L*).

These data indicate that many diverse repetitive lysine-rich sequences, in which the size of the repeating unit can vary from 3 to 30 residues in length, have a propensity to localize to the periphery of the infected erythrocyte. Of the 11 repetitive sequences with a lysine content >20% that were tested, nine were localized to the erythrocyte periphery. Although many of the repeating sequences contain both acidic and basic residues, most sequences capable of targeting the erythrocyte periphery had a theoretical isoelectric point value of >9 (Table 1). The two exceptions, MESA (pI of fragment, 4.90) and the PHISTb/c protein PF3D7_1476200 (pI of fragment, 4.71), both display the least prominent peripheral targeting, and the aspartate-rich repeats of PF3D7_0114200 (pI 5.12) remained entirely cytosolic. Acidic residues may therefore interfere with the peripheral localization of some lysine-rich repeating sequences.

**FIGURE 5.**
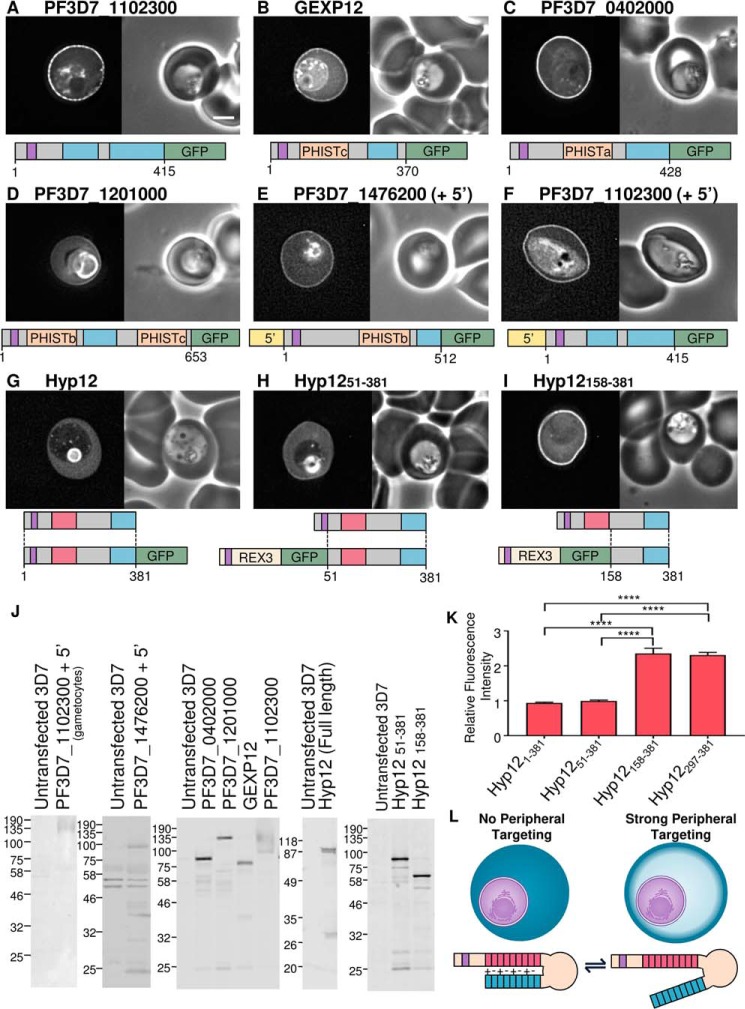
**Localization of lysine-rich repeat-containing proteins.** *A–I*, identities of proteins are shown *above* each image. Shown are GFP localization (*left*) and a phase-contrast image (*right*). A representation of the full-length protein is shown *below* each image, with the lysine-rich repeat regions in *blue*, export sequences (signal sequence and PEXEL/HT motif) in *purple*, PRESAN/PHIST domains in *orange*, and acidic regions in *red*. Expressed fragments are also illustrated *below G–I. Scale bar*, 2 μm. *J*, anti-GFP Western blots of all parasite lines. *K*, ratio of the fluorescence intensity at the erythrocyte periphery relative to the erythrocyte cytoplasm, as in previous figures. ****, an extremely significant difference (*p* ≤ 0.0001). *L*, model showing the potential masking of the C-terminal lysine-rich sequence by the acidic N terminus of Hyp12. *Error bars*, S.E.

Having determined that lysine-rich repetitive sequences, when fused to GFP, can localize to the periphery of the infected erythrocyte, we next tested whether these sequences function similarly in the context of the corresponding full-length proteins. GFP-tagged PF3D7_1102300, GEXP12, PF3D7_0402000, and PF3D7_1201000 were expressed, and all showed peripheral localization (Fig. 5, *A–D*, respectively). PF3D7_1201000 showed a very weak localization to the cell periphery, which is similar to the localization of the isolated lysine-rich fragment; GFP fluorescence was also accumulated in the parasitophorous vacuole in this case. LYMP, MESA, and KAHRP have previously been localized to the periphery of infected cells by immunofluorescence (39, 45, 59, 60). GFP-tagged PF3D7_1476200, when expressed from the calmodulin promoter, has previously been localized to the periphery of the infected erythrocyte (43). When expressed from its own promoter, the protein localization is similar (Fig. 5*E*). Similarly, GARP, when expressed from its own promoter, is also peripherally localized (Fig. 1*J*). Detection by Western blotting of this protein is variable; smeared bands and prominent fragments of the protein are often detected (Fig. 1*K*). Transcripts of GEXP12 and PF3D7_1102300 are enriched in gametocyte stage parasites relative to the asexual stage (61). To test whether lysine-rich sequences can also target proteins to the erythrocyte periphery in this life cycle stage, we expressed GFP-tagged PF3D7_1102300 from its own promoter. Within a mixed culture, most brightly GFP-expressing parasites were gametocytes (Fig. 5*F* and supplemental Fig. 1, *AJ*). The GFP localization is consistent with the protein targeting to the periphery of the gametocyte-infected cell. Expression of proteins was confirmed by Western blotting (Fig. 5*J*). Gametocyte-enriched parasites were purified for Western blots of PF3D7_1102300, which was detected as a smeared band at a molecular weight higher than expected. Other proteins were detected at approximately the expected sizes.

##### The Targeting Function of the Lysine-rich Sequence in Hyp12 Is Masked by an Acidic Sequence

We also localized GFP tagged full-length Hyp12 protein. The lysine-rich fragment of Hyp12 is efficiently recruited to the periphery of the red cell (Fig. 4*I*). By comparison, the full-length protein with either a C- or N-terminal GFP tag is not efficiently recruited to the cell periphery (Fig. 5, *G* and *H*, respectively). This localization for the full-length protein has also been described previously (62). Hyp12 contains a C-terminal lysine-rich sequence but also a highly acidic N-terminal sequence. The acidic sequence is also repetitive and is predicted to be intrinsically disordered. To test the possibility that this sequence is able to inhibit the targeting function of the lysine-rich sequence, the C terminus of Hyp12 protein lacking the acidic sequence was expressed. This protein is robustly recruited to the cell periphery, suggesting that the acidic sequence masks the targeting function of the lysine-rich sequence within this protein (Fig. 5, *I*, *K*, and *L*).

##### Variation in Length between Lysine-rich Repeat Regions in Different P. falciparum Strains

The length of repeat sequences often varies between different parasite strains (63), and the preceding experiments suggest that variation in length of lysine-rich repeats may influence the efficiency with which these sequences can target proteins to the erythrocyte periphery. To determine the extent of repeat length variation seen in lysine-rich repeat sequences, we analyzed sequences from the genomes of several laboratory strains of parasites (3D7, DD2, HB3, IT, and 7G8) as well as 11 parasites isolated from infected people from diverse geographic locations (“long read” sequence data generated by the Pf3k consortium was used for these analyses to ensure the correct assembly of repetitive regions).

**FIGURE 6.**
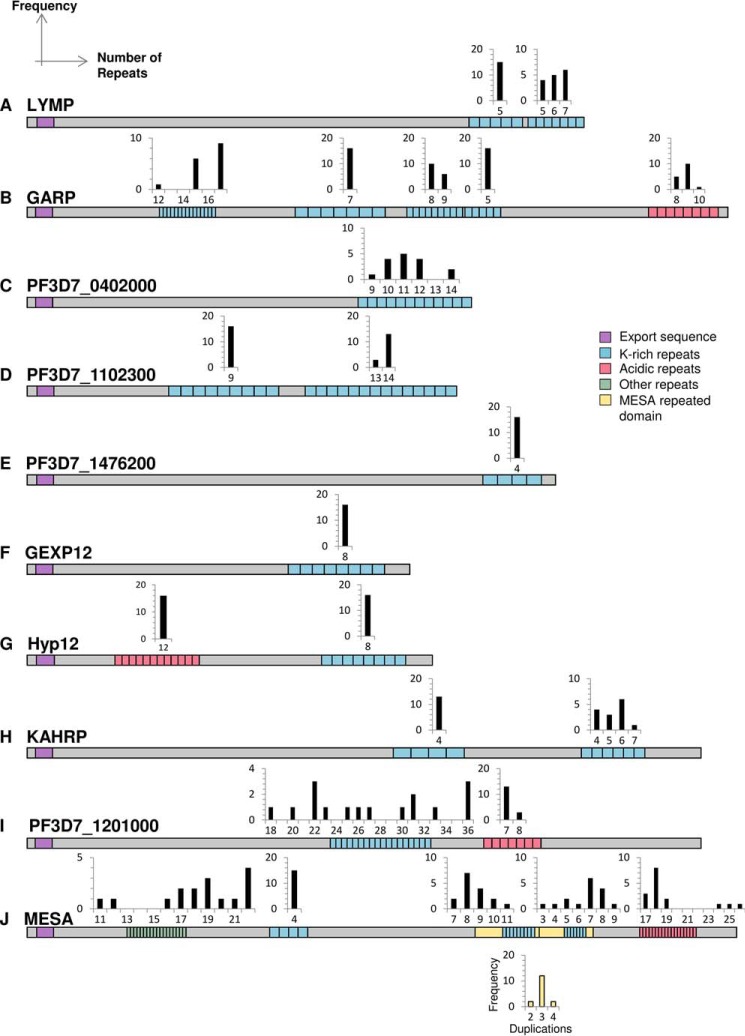
**The number of repeating units in periphery-targeting proteins varies between different field isolates and laboratory strains.** *A–J*, alignment of proteins containing lysine-rich repeating regions indicates considerable variation in length between strains. Histograms represent the frequency (*y* axis) with which a given number of repeats (*x* axis) is observed in a repeat region. Schematics show the repeat architecture of the proteins from the PF3D7 strain. The export sequences (signal sequence and PEXEL/HT motif) are shown in *purple*, lysine-rich repeats in *blue* (>20% lysine), acidic repeats in *red*, other repeats in *green*, and the duplicated MESA domain in *yellow*. A histogram indicating the number of duplications of the MESA domain is shown *below* the protein with *yellow bars*. Schematics are approximately to scale, with MESA downscaled by one-half.

Significant variation in repeat number is seen in many lysine-rich targeting sequences. The C-terminal repeating sequence of LYMP, the first repeat of GARP, and the PHISTa protein PF3D7_0402000 contain 5–7, 12–17, and 9–14 copies of repeating motifs, respectively (Fig. 6, *A–C*). Although unlikely to lead to a complete loss or gain of peripheral localization, these changes may modulate the targeting efficiency of these protein sequences. The C-terminal repeat region of PF3D7_1102300 contains either 13 or 14 copies of the repeat motif EREKREKKEKE, but the repeat sequences of PHISTB protein PF3D7_1476200, PHISTc protein GEXP12, and Hyp12 are invariant (Fig. 6, *D–G*). The 5′ repeats of KAHRP do not vary, but variations in repeat number are observed for the 3′ repeats (Fig. 6*H*), as has been reported previously (64, 65). In 3D7 parasites, the protein PF3D7_1201000 contains two PRESAN domains, which are separated by 18 units of the sequence DEKEK. In all other parasites, the repeat unit number has increased, in some cases by as much as 2-fold (Fig. 6*I*).

In 3D7 parasites, MESA contains five repeat sequences; all except for the second repeat sequence vary significantly in length. In the 3D7 genome, the sequence encoding the third repeat region, which is itself variable in length (Fig. 6*J*), is duplicated to form the fourth repeat. In other genomes, the sequence is further duplicated, resulting in three or four copies of this repeat sequence and its flanking regions. GFP-tagged lysine-rich sequences from both MESA and PF3D7_1201000 display a weak fluorescence signal at the erythrocyte periphery, and duplication and extension of the repeat regions may increase the targeting efficiency of these sequences.

##### Peripheral Targeting of Lysine-rich Repeating Sequences Is Conserved between Plasmodium Species

To investigate whether the targeting of lysine-rich repeat regions to the erythrocyte periphery is conserved, we also searched other parasite genomes for putative exported proteins that contain lysine-rich repeating sequences. The proteins predicted to contain sequences with a targeting function are shown in supplemental Table 1. The largest numbers of potential periphery-targeting sequences were found in the *P. reichenowi* genome, with 20 proteins containing lysine-rich repeats, most of which are syntenic to those identified in *P. falciparum*. The genomes of three closely related species that infect primates, *Plasmodium knowlesi*, *Plasmodium vivax*, and *Plasmodium cynomolgi*, contained 19, 15, and 6 proteins containing lysine-rich repetitive regions, respectively, whereas fewer sequences were predicted for *Plasmodium* species infecting rodents; *Plasmodium yoelii*, *Plasmodium chabaudi*, and *Plasmodium berghei* (supplemental Table 1).

**FIGURE 7.**
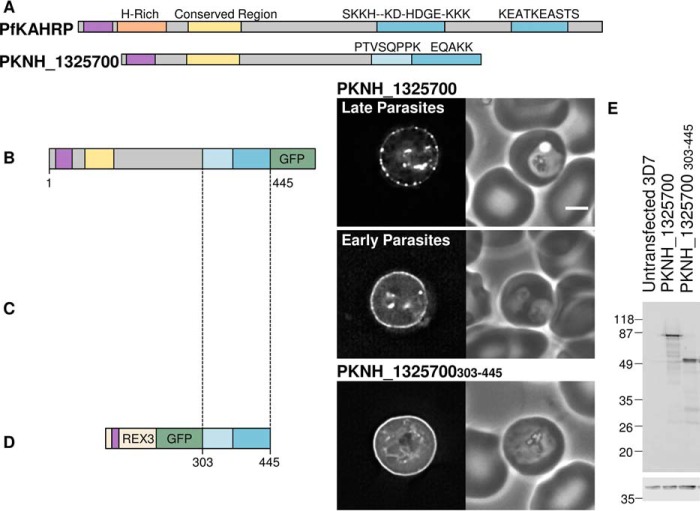
**The *P. knowlesi* protein PKNH_1325700 contains a C-terminal periphery-targeting repetitive sequence and an N-terminal domain also found in PfKAHRP.** *A*, representation of *P. falciparum* KAHRP (*top*) and *P. knowlesi* protein PKNH_1325700 (*bottom*), with lysine-rich repeat regions shown in *blue* and their consensus motifs shown *above*. The first repeat of PKNH_1325700 contains 12.5% lysine residues and is *colored light blue*. The conserved region found in both proteins is shown in *yellow*, the histidine-rich region in *orange*, and the export sequence in *purple. B* and *C*, *P. falciparum* parasites expressing the GFP-tagged full-length PKNH_1325700 in late parasites and early parasites, respectively. *D*, GFP-tagged C-terminal repeat region of PKNH_1325700. A schematic of the protein, a GFP fluorescence image, and a phase-contrast image are shown from *left* to *right. Scale bar*, 2 μm. *E*, Western blots with anti-GFP (*top*). Anti-HAP was used to confirm equal loading (*bottom*).

To test whether lysine-rich sequences from parasites other than *P. falciparum* have targeting functions, we tested the localization of the *P. knowlesi* protein PKNH_1325700 in *P. falciparum*-infected erythrocytes. This protein contains a PEXEL/HT sequence, RSLSV, and two repetitive lysine-rich stretches at its C terminus (Fig. 7*A*). Full-length PKNH_1325700 was efficiently exported to the erythrocyte, where the GFP signal appears as a partially punctate distribution around the periphery of the red blood cell (Fig. 7*B*). In younger parasites, fewer of these puncta were present, and a continuous line of fluorescence was apparent around the periphery of the cell (Fig. 7*C*). To test whether the lysine-rich sequence alone is able to target to the erythrocyte periphery, REX3 and GFP were fused to residues 303–445 of PKNH_1325700; this includes the first and second lysine-rich repeat regions, which have lysine contents of 12.5 and 40%, respectively. This GFP-tagged protein formed a continuous ring at the erythrocyte periphery (Fig. 7*D*), indicating that lysine-rich sequences from multiple parasite species can form modules with a targeting function. Anti-GFP Western blotting confirmed the expression of proteins at the expected size (Fig. 7*E*).

##### A Conserved Protein Family Containing an EMP3-KAHRP-like Domain and Expanded Repeated Sequences

**FIGURE 8.**
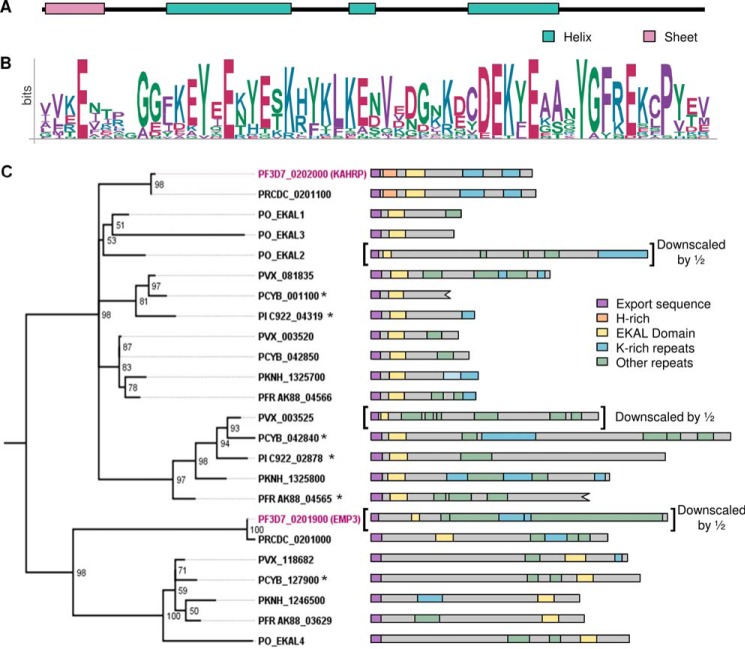
**The EKAL domain is present within multiple repeat-containing *Plasmodium* proteins.** *A*, secondary structure prediction for the EKAL domain of PfKAHRP (PSIPRED (85)). *B*, sequence logo of the EKAL domain derived from all 24 proteins. Residue position is shown on the *x* axis, and conservation is represented on the *y* axis (*bits*). *C*, *left*, phylogenic tree of KAHRP and EMP3 homologs in *P. falciparum* (*Pf3D7*), *P. reichenowi* (*PRCDC*), *P. vivax* (*PVX*), *P. knowlesi* (*PKNH*), *P. cynomolgi* (*PCYB*), *P. fragile* (*PFR*), *P. inui* (*PI*), and *P. ovale* (*PO*). Proteins that may contain frameshift mutations are indicated with an *asterisk* (see supplemental Table 2*B*). *P. ovale* proteins were assembled *de novo* and have been named EKAL1–4. *P. fragile* and *P. inui* proteins are named according to their assigned gene names preceded by *PFR* or *PI*, respectively. *Numbers* at each node represent quartet puzzling (*QP*) support values predicted by TREEPUZZLE, where values represent the reliability of groupings (118). *Right*, diagrams representing each protein sequence, with EKAL domains in *yellow*. Export sequences are shown in *purple* (signal sequence and PEXEL/HT motif). Many proteins contain lysine-rich tandemly repeated sequences (*blue*) as well as repeating sequences that do not contain >20% lysine (*green*). The first repeating sequence of PKNH_1325700 is shown in *light blue* because only 12.5% of residues are lysine. The histidine-rich regions of *P. falciparum* and *P. reichenowi* KAHRP are shown in *orange*. Schematics are approximately to scale, with PVX_003525, Pf3D7_0201900, and PO_EKAL2 scaled down by half. PCYB_001100 and PFR A0A0D9QJA3 sequences are truncated due to gaps in the assembled sequences.

**FIGURE 9.**
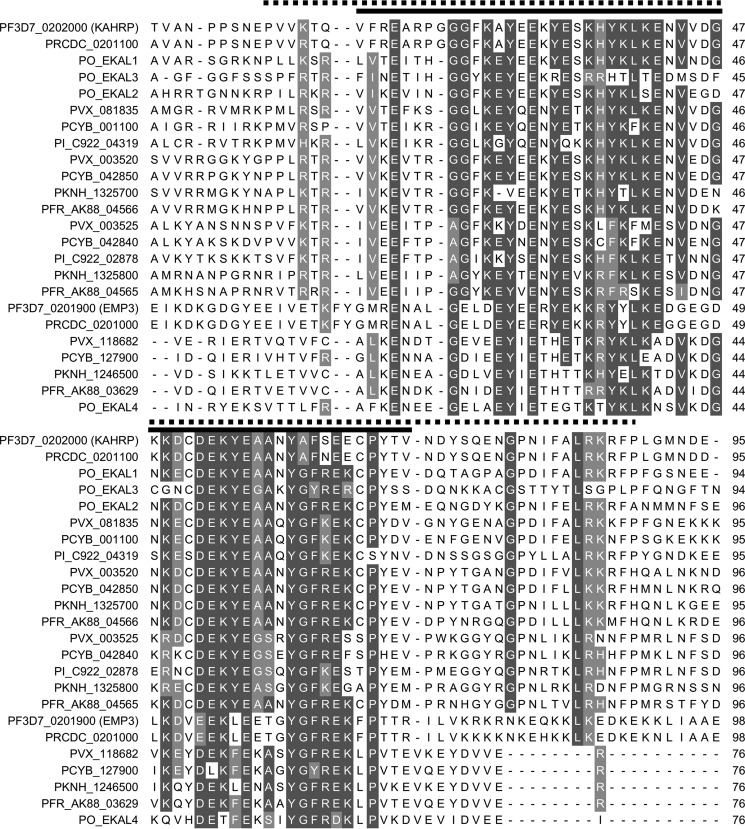
**Alignment of the EKAL domain in proteins identified as homologs of KAHRP and EMP3 in *P. falciparum*, *P. reichenwowi*, *P. knowlesi*, *P. vivax*, *P. cynomolgi*, *P. fragile*, *P. inui*, and *P. ovale*.** Proteins were aligned using T-COFFEE (113). Residues with >70% identity or similarity are *shaded* in *dark gray* and *light gray*, respectively, using Multiple Align Show (114). A *black line above* the alignment represents the highly conserved EKAL domain, and a *dotted line* represents an extended conserved domain used for assembling phylogenic trees.

Notably, PKNH_1325700 also contains an N-terminal 70-residue sequence, which is predicted to form a folded domain (Fig. 8*A*) and is homologous to the N terminus of *P. falciparum* KAHRP (41). Although the repeating motifs found in the C-terminal sequences of PKNH_1325700 and KAHRP are not related, they are similar in that they are lysine-rich, and both sequences target to the erythrocyte periphery. The presence of an N-terminal conserved domain and C-terminal lysine-rich repeating sequences in both KAHRP and PKNH_1325700 suggests that these proteins may to some extent be functionally related. Given that KAHRP is a key cytoskeleton-associated protein involved in sequestration of *P. falciparum*-infected erythrocytes, we searched for proteins that have similar domain architecture in other species. In *P. falciparum*, the conserved N-terminal domain is also found at the N terminus of the erythrocyte cytoskeleton-associated PfEMP3 protein; the remainder of this protein is also formed of repeating sequences, including a central lysine-rich region (Fig. 8*C*). Because the domain is present in both EMP3 and KAHRP, we refer to it as the EMP3-KAHRP-like (EKAL) domain. KAHRP-like proteins have previously been identified in some species (41); we identify additional EKAL domain-containing proteins in the genomes of the primate-infecting parasites *P. reichenowi*, *P. knowlesi*, *P. vivax*, *P. cynomolgi*, *Plasmodium fragile*, *Plasmodium ovale*, and *Plasmodium inui* (Figs. 8 (*B* and *C*) and 9). These proteins can be grouped into seven branches; five branches are closely related to PfKAHRP, whereas two represent homologs of the EMP3 protein (Fig. 8*C*). Remarkably, each parasite genome encodes at least one protein with a KAHRP-like EKAL domain that is followed by a C-terminal lysine-rich repeating sequence that may target the protein to the periphery of the infected host cell (Fig. 8*C*). Although sequence homology in PfEMP3- and KAHRP-like proteins is largely restricted to the EKAL domain, it is likely that in many cases, the expansion of divergent repetitive lysine-rich sequences has generated protein modules that contribute to the peripheral localization of this protein family in the infected erythrocyte.

## Discussion

Repetitive sequences in many organisms are crucial for protein function (66–73) (reviewed in Ref. 4), yet there are currently few functions assigned to repeats in *Plasmodium*. We show that several proteins from *P. falciparum* contain lysine-rich tandemly repeating sequences that confer a peripheral localization in the infected erythrocyte. Four of the nine proteins identified were previously uncharacterized, including GARP, which contains three distinct lysine-rich repeat sequences with a targeting function.

The rapid expansion and contraction of repeating sequences suggests that they can contribute significantly to protein evolution and the generation of novel functional modules (4, 63, 74, 75). Within PfGARP, decreasing the number of repeating units within the N-terminal lysine-rich sequence proportionally decreases the efficiency of targeting. Given this, it is likely that exported parasite proteins can rapidly evolve novel localization domains by expanding short low affinity lysine-rich motifs to create high avidity targeting sequences. Comparison of the repeating sequences of *P. falciparum* GARP with those found in GARP from *P. reichenowi* and *P. gaboni* provides two examples of such repeat expansion occurring. In the first repeating sequence of *P. falciparum* GARP, the repeat EKK has expanded to generate a periphery-targeting sequence, whereas in *P. reichenowi*, a more acidic repeat has expanded, which does not efficiently localize to the periphery.

**FIGURE 10.**
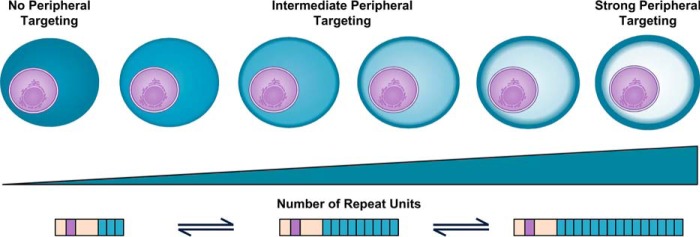
**Evolution of novel targeting domains and modulation of targeting efficiency by repeat expansion.**

Smaller changes in repeat number may also subtly modulate the targeting efficiency of lysine-rich repeating sequences (Fig. 10). Within proteins that modulate key properties of the host cell, such as rigidity, cytoadhesion, and nutrient import, such changes could confer a selective advantage. Indeed, correlation between repeat sequence length and phenotype has been observed in other organisms (68–70), and the number of repeat motifs within the functionally important C-terminal domain of *P. falciparum* RNA polymerase II also varies between isolates (76). Analysis of lysine-rich targeting sequences from laboratory and field strains of *P. falciparum* parasites confirms that repeat units can be both lost and gained from these sequences. There is a high level of conservation between repeat motifs within targeting sequences; this is a common feature of disordered repetitive sequences and suggests that the repeats were recently expanded and may be particularly dynamic (77, 78). This may allow rapid adaption of parasites under selective pressure.

Although Hyp12 contains a lysine-rich sequence that targets the cell periphery, the targeting function is masked by an acidic repetitive sequence. Expansion or contraction of either sequence in Hyp12 could lead to a change in protein localization. Contraction of the acidic sequence might reduce the inhibitory propensity of this sequence, whereas expansion of the lysine-rich sequence might allow it to overcome the inhibition by the acidic sequence. Over evolutionary time, the localization of this protein may be determined by two “competing” repetitive, low complexity, disordered sequences. It remains unclear whether there is a physiological stimulus that might unmask the lysine-rich sequence in Hyp12; proteolytic cleavage or changes in ionic composition or temperature could potentially regulate this process. Notably, deletion of the gene encoding Hyp12 leads to a change in infected cell rigidity (79).

Targeting of proteins by lysine-rich repeating sequences is not restricted to *P. falciparum* proteins. The protein encoded by the *P. knowlesi* gene PKNH_1325700 contains an N-terminal EKAL domain, with homology to the N terminus of *P. falciparum* KAHRP (41), and two adjacent lysine-rich repeat sequences at its C terminus. Although the repeated motifs differ from those in *P. falciparum* KAHRP, the lysine-rich repeats of both proteins localize uniformly to the erythrocyte periphery. The full-length PKNH_1325700 protein, however, appears as a number of peripherally located disperse dots, suggesting that the N terminus is prone to self-association. KAHRP is a key component of the electron-dense cytoadherence-related knob structures that are seen in *P. falciparum* infected cells and that are also observed in *P. fragile*-infected rhesus monkey erythrocytes (80). However, although *P. vivax*- and *P. knowlesi*-infected cells adhere to specific ligands (81–83), knoblike structures are not seen on erythrocytes infected with these parasites. In addition to PKNH_1325700, we find at least one KAHRP-like gene characterized by an EKAL domain and a repetitive lysine-rich sequence in the genomes of *P. reichenowi*, *P. vivax*, *P. ovale*, *P. cynomolgi*, *P. fragile*, and *P. inui*. Knob structures cluster PfEMP1 proteins in *P. falciparum*-infected cells. Although parasites other than *P. falciparum* and *P. reichenowi* do not express PfEMP1 proteins, other variant surface antigens have been identified in other species (84); it is possible that the KAHRP homologues in these species play a role in clustering of these proteins on the surface of infected cells in structures that are not morphologically distinctive or electron-dense. Notably, EKAL domains and repeating sequences are also found in PfEMP3 and its homologues. Like KAHRP, PfEMP3 is involved in PfEMP1 trafficking, localizes to the Maurer's clefts and cytoskeleton of infected cells, and affects infected cell rigidity (23, 25, 26, 48). Expansion of different repeat sequences may represent a means of diversifying the function of EKAL domain-containing proteins.

Although several of the identified lysine-rich targeting sequences are found in proteins with known interacting partners, the identity of the binding partner of the lysine-rich sequences remains unclear. We show that a fragment of KAHRP encompassing the 5′ lysine-rich repeats is sufficient to target to the erythrocyte periphery *in vivo*. This region is important for the cytoadhesion-modulating function of the protein (38); however, the binding partners of the KAHRP repeating sequences remain controversial. It has been suggested that the 5′ lysine-rich repeat region interacts with PfEMP1 (86, 87), but this interaction was not observed in other studies (88). Fragments of KAHRP that include the 5′ lysine-rich repeat sequence also bind to spectrin *in vitro* (89). Although the repeat sequence alone was not sufficient for this interaction under previous experimental conditions (89, 90), recent work indicates that the 5′ repeats are sufficient for spectrin binding.^3^ *In vitro*, the C terminus of LYMP interacts with inside out erythrocyte vesicles (39) and with purified band 3 (44). It is unclear whether the lysine-rich repeats, which are located in the final 100 residues of this fragment, contribute to this interaction, but a fragment comprising only the lysine-rich repeats of LYMP does not bind to inside-out erythrocyte vesicles *in vitro* (39). In MESA, the lysine-rich sequence shown here to localize to the erythrocyte periphery was also shown to be insufficient for binding inside-out erythrocyte membranes (51). This may indicate that these lysine-rich repeats interact with *Plasmodium* proteins or cytoskeletal components that are post-translationally modified during infection (91, 92). Given the diversity of lysine-rich repeat sequences that can target to the erythrocyte periphery, it is possible that they interact with different host or parasite proteins.

Several proteins that contain lysine-rich targeting sequences also contain other well characterized cytoskeleton-targeting domains, suggesting that they cross-link multiple components of the erythrocyte cytoskeleton or membrane. Indeed, LYMP functions by linking PfEMP1 and band 3 via its PRESAN domain and C terminus, respectively (44, 45). A lysine-rich repeating C terminus is also seen in other proteins with PRESAN domains capable of targeting the periphery, including PF3D7_0936800 (45) and PF3D7_1476200 (43). Two other uncharacterized proteins with peripherally localized lysine-rich repeating sequences also contain PRESAN domains: the PHISTC protein GEXP12 and PF3D7_1201000, which contains N- and C-terminal PRESAN domains from the PHISTb and -c families, respectively. It is possible these proteins play roles similar to that of LYMP at the erythrocyte periphery. However, not all PRESAN domains interact with PfEMP1; the PHISTa protein PF3D7_0402000 binds to band 4.1 (52). Both PF3D7_0402000 and MESA contain lysine-rich repeat sequences capable of associating with the erythrocyte periphery in addition to band 4.1-binding domains. Although previous immunofluorescence experiments suggest that PF3D7_0402000 co-localizes with band 4.1, a significant fraction of the protein was localized in the parasitophorous vacuole. This is not consistent with the localization that we observe for the GFP-tagged protein; it is possible that the antibody epitope is hidden when the protein is bound to the erythrocyte cytoskeleton (52).

The proteins GARP and PF3D7_1102300 are predicted to be entirely intrinsically disordered, and repeating sequences make up 44 and 66% of the mature proteins, respectively. It is therefore possible that the interaction of the lysine-rich sequences with their target fulfills the function of the protein. Interestingly, expression of GARP is up-regulated in parasites isolated from children with severe malaria (93), and GARP is differentially expressed in parasites selected for adherence to different ligands (94). PF3D7_1102300 is up-regulated during heat shock (40) and also in parasites selected for cytoadhesion (95). Deletion of the genes encoding GARP and PF3D7_1102300 as well as the PHISTa protein PF3D7_0402000 and PHISTb/c protein PF3D7_1201000 does not result in a striking phenotype; however, some decrease in infected cell rigidity is observed (79). Given the similarity between many of the lysine-rich proteins that we have characterized, it is likely that individual genes may be functionally redundant and that deletion of single genes may not be sufficient to reveal a phenotype (79).

Some proteins may also function in the gametocyte stage, during which the rigidity of the infected cell changes (96). GEXP12 transcripts and peptides are detected in both asexual stage parasites and gametocytes (97, 98). Because we have used the calmodulin promoter to express GFP-tagged GEXP12, we are only able to assess its localization in asexual stages; this shows that the protein has a propensity to localize to the erythrocyte periphery. Notably, when GFP-tagged PF3D7_1102300 is expressed from its own promoter, the protein is localized to the periphery of gametocyte-infected cells, indicating that proteins containing lysine-rich sequences can also be similarly targeted during this life cycle stage. Given this, it might be expected that GEXP12 would also localize to the cell periphery in the gametocyte stage.

Electrostatic interactions between the basic lysine residues and a negatively charged surface, either protein or lipid, are probably responsible for the peripheral localization of the repeating sequences. Other basic residues may confer a similar localization. A polyhistidine sequence in KAHRP also targets the erythrocyte periphery (58); however, arginine residues are underrepresented in the AT-rich parasite genome (3). Interestingly, despite the high predicted isoelectric points of most of the sequences, many peripherally localized repeats also contain acidic residues, and targeting does not appear to require a strict sequence consensus or repeat length. This makes accurate prediction of sequences with a targeting function difficult. Two lysine-rich proteins tested did not associate with the erythrocyte periphery, and whereas some untested proteins, such as the FIKK kinases FIKK4.1 and FIKK7.1 and the megadalton repeat protein Pf11-1, are implicated in modulating cytoskeletal properties (91, 99, 100), FIKK4.1 and the PfEMP1 trafficking protein (PTP3) have been shown to localize to Maurer's clefts or the erythrocyte cytoplasm, respectively (79, 101).

The observation that repetitive lysine-rich sequences in *Plasmodium* can target proteins to the periphery of the infected erythrocyte suggests that such proteins will perform key functions at the host parasite interface. Moreover, the potential for expansion and contraction of these sequences to modulate targeting efficiency or to generate novel targeting sequences suggests that they play important roles in evolution of proteins targeted into the host erythrocyte.

## Experimental Procedures

### 

#### 

##### Plasmids and Parasite Transfection

Gene sequences were amplified from *P. falciparum* (3D7), *P. knowlesi* (A1H.1), or *P. reichenowi* genomic DNA and inserted into *P. falciparum* expression plasmids containing an attP site. Gene expression was controlled by the *P. falciparum* calmodulin promoter and *P. berghei* dihydrofolate reductase-thymidylate synthase 3′-untranslated region. Gene sequences encoding full-length proteins were cloned in frame with 3′ GFP and STREPII tags. Constructs with 5′ truncations were fused to a sequence encoding the N-terminal 61 residues of PFI1755c (REX3); this sequence contains the N-terminal signal sequence and PEXEL/HT motif of REX3. These plasmids contained REX3(1–61), GFP, a linker sequence (LESGSGTGASDV), and the lysine-rich sequence-encoding fragment, followed by a STREPII tag. The linker was not included in the following constructs shown in Fig. 1: GARP(50–118), GARP(119–163), GARP(253–340), GARP (372–446), and GARP(535–673). All cloned *P. falciparum* sequences matched the 3D7 genome sequence; however, two silent base pair mutations were made in the sequences of GARP(134–163) and GARP(149–163) to facilitate cloning through overlap PCR. The *P. knowlesi* gene PKNH_1325700 contained an insertion corresponding to one repeat of the KKEQA motif in both the full-length and truncated constructs. The *P. gaboni* GARP fragment was constructed *de novo* using multiple primers based on a DNA sequence assembled from multiple short sequencing reads (see below for details). Full-length GARP, PF3D7_1102300, and PF3D7_1476200 were also expressed under their own promoters; the PfCAM promoter was replaced with sequences starting 932, 967, and 1084 bp upstream of the start codon for each gene, respectively.

Expression plasmids, together with a plasmid encoding the Bxb1 integrase (102), were transfected (103) into a 3D7attB parasite strain (obtained through BEI Resources, NIAID, National Institutes of Health: *P. falciparum* 3D7-attB, MRA-845, deposited by D. A. Fidock) (104). Transfected parasites were selected using 2.5 nm WR99210 and 2 μg/ml blasticidin.

##### Microscopy

Parasites were fed 1 day before imaging. A drop of culture material in RPMI was placed between a microscope slide and coverslip. Phase-contrast and GFP fluorescence images were acquired at room temperature with a Zeiss Axiovert 200M microscope equipped with an HBO100 lamp and a ×100 oil lens with a numerical aperture of 1.30. Images were taken with an AxioCam MR camera using AxioVision software release 4.8.2. Z-stacks of images were collected and deconvolved by iterative restoration (confidence limit, 95%; iteration limit, 10) using Volocity; a single image from the Z-stack is presented. Images were cropped, and automatic brightness and contrast settings were applied using ImageJ.

##### Statistical Analysis

The average fluorescence intensity at the periphery relative to the cytoplasm of infected cells was quantified in ImageJ as described in supplemental Fig. 1. Statistical analysis was performed in GraphPad Prism version 7 using ordinary one-way analysis of variance with each parasite line compared with the GFP-tagged REX3(1–61) fragment to establish whether proteins were significantly enriched at the erythrocyte periphery. Fisher's uncorrected least significant difference test was used for multiple comparisons. *p* values are reported in Table 2. *Labels* represent significance. ns, *, **, ***, and **** indicate not significant (*p* > 0.05), *p* ≤ 0.05, *p* ≤ 0.01, *p* ≤ 0.001, and *p* ≤ 0.0001, respectively.

##### Western Blotting

Schizonts were purified using 70% Percoll, 3% sorbitol in PBS (105). Approximately 5 × 10^6^ schizonts were loaded per lane. Blots were probed with rabbit α-GFP antibody (Torrey Pines, catalogue no. TP401, lot 071519) diluted 1:4000 or mouse α-histoaspartic protease (HAP) (obtained through BEI Resources, NIAID, National Institutes of Health: monoclonal antibody 3F10-6 anti-*P. falciparum* HAP, MRA-811A, contributed by Daniel E. Goldberg) antibody diluted 1:1000, as indicated (106). Goat anti-rabbit (Thermo Fisher Scientific, catalogue no. 35568, lot OK195926) and goat anti-mouse (Thermo Fisher Scientific, catalogue no. 35521, lot LB143097) secondary antibodies were diluted 1:10,000. Membranes were imaged with a LI-COR Odyssey imager. The specificity of the α-GFP antibody was confirmed by Western blotting of parasites not expressing GFP. For Western blotting of parasites expressing PF3D7_1102300 under its own promoter, gametocytes were enriched and purified as described previously (107).

##### Comparison of GARP Genes from Closely Related Parasite Species

A GARP homologue from *P. gaboni* was assembled from two incomplete protein coding sequences deposited in the NCBI (GenBank^TM^ accession numbers KYN95113.1 and KYN95116.1); the connecting region was assembled from sequence reads (GenBank^TM^ biosamples SAMN04053641 and SAMN04053639) (57). The *P. reichenowi* GARP gene sequence (PRCDC_0111200) was from PlasmoDB (version 26).

##### Identification of Putative, Exported, Lysine-rich, Repeating Protein Sequences

Protein coding sequences from *P. falciparum*, *P. vivax*, *P. knowlesi*, *P. cynomolgi*, *P. reichenowi*, *P. berghei*, *P. chabaudi*, and *P. yoelii* (*17X*) were downloaded from PlasmoDB (version 26). Putative exported proteins were identified by the presence of either a signal sequence (defined by SignalP (108)) or a transmembrane domain within the first 100 residues (defined using MPEX translocon TM analysis (109)) and an R*X*L motif in the 50 residues following the signal sequence/transmembrane segment. Proteins containing more than four transmembrane segments within the coding sequence are unlikely to be exported and were excluded from further analysis.

A custom perl script utilizing a sliding window algorithm was used to identify proteins containing stretches of amino acids of ≥30 residues in length with with a lysine content of ≥20%. Within the set of lysine-rich sequence fragments, repeating protein sequences were identified using the tandem repeat predictor XSTREAM (110). Parameters for XSTREAM were as follows: minimum word match = 0.6, minimum consensus match = 0.6, maximum period = 30, miss penalty = −3, and gap penalty = −3 (111). Another custom perl script was used to interpret the output of XSTREAM and select proteins in which the sequence region composed of repeats was >30 residues in length. Multiple lysine-rich repeat sequences were found in some proteins. XSTREAM was used to define the consensus sequence of each repeated array, the consensus error value for each repeat array, and the position of the repeated array within each protein. More stringent parameters were used to reduce the number of gaps in the consensus sequence, with minimum word match = 0.6, minimum consensus match = 0.65, miss penalty = −3, and gap penalty = −5. Maximum period value was set to 30 residues unless shorter repeats were apparent within the predicted consensus sequence; other parameters were set to default values. The consensus sequences of the degenerate repeats of Hyp12 and PF3D7_0106600 were defined using less stringent criteria. Theoretical isoelectric point values were predicted by PROTPARAM (112).

##### Sequence Analysis of Proteins from Different Parasite Isolates

Protein sequences of lysine-rich proteins from different *P. falciparum* parasite strains were extracted from unassembled long read PACBIO genome sequencing data obtained from the Pf3k consortium. Five laboratory isolates were included (3D7, DD2, IT, 7G8, and HB3) as well as 11 field isolates from Gabon, Guinea, United Kingdom, Kenya, Mali, Sudan, Senegal, Democratic Republic of the Congo, Togo, and Cambodia. No KAHRP genes were found in the DD2 or Kenyan isolates. LYMP was not found in one of the two Cambodian isolates. All alignments were created with T-COFFEE (113) and represented with “Multiple Align Show” (114). 10 of 141 gene sequences, indicated in supplemental Table 2*A*, contain frameshift point mutations. It is unclear whether these represent genuine mutations or sequencing errors in database sequences; for the purpose of sequence alignment, the reading frames were restored (see supplemental Table 2*A*).

##### Sequence Analysis of the KAHRP Conserved Domain

Proteins with homology to the conserved domain of KAHRP and PfEMP3 were identified by HMMer (115). Additionally, homologous sequences within the *P. ovale* genome were identified from unassembled sequence reads acquired from the Sanger Institute through the use of the in-built BLAST server. Sequence reads from *P. ovale* containing EKAL domains were assembled using the SEQman Ngen software (116). Introns were manually annotated within genes from *P. ovale*, *P. fragile*, *P. inui*, and *P. cynomolgi* where necessary. Potential sequencing errors resulting in frameshift mutations were corrected, and introns were annotated based on known *Plasmodium* splice sites. These modifications were made in five proteins from *P. inui* and *P. cynomolgi* (see supplemental Table 2*B* for details). Sequences were aligned with T-COFFEE (113) in Jalview (117). Maximum likelihood estimation with TREE-PUZZLE (118) was used to create phylogenic trees based on an extended conserved domain (see Fig. 9 for details), which were assembled with FigTree version 1.2.4 (119). Secondary structure predictions and disorder predictions were made by PSIPRED (85) and DISOPRED (55), respectively.

## Author Contributions

Experiments were conducted by H. M. D. and A. R. O. and designed by H. M. D., K. T., and A. R. O. H. M. D. and A. R. O. wrote the paper, and all authors approved the final version of the manuscript.

## Supplementary Material

Supplemental Data

## Footnotes

3 E. E. Cutts, N. Laasch, D. M. Reiter, R. Trenker, L. M. Slater, P. J. Stansfeld, and I. Vakonakis, personal communication.

## Footnotes

2 The abbreviations used are:

PEXEL: *Plasmodium* export element
PRESAN domain: *Plasmodium* RESA N-terminal domain
PHIST: polyhelical interspersed subtelomeric
HT: host-targeting
KAHRP: knob-associated histidine-rich protein
GARP: glutamic acid-rich protein
MESA: mature parasite-infected erythrocyte surface protein
LYMP: lysine-rich membrane-associated PHISTb
RESA: ring-infected erythrocyte surface protein
PACBIO: Pacific Biosciences sequencing
EKAL: EMP3-KAHRP-like domain
HAP: histoaspartic protease.
