# Supplementary material for: Expansion of Lysine-rich Repeats in Plasmodium Proteins Generates Novel Localization Sequences That Target the Periphery of the Host Erythrocyte
Source: J Biol Chem. 2016 Oct 24;291(50):26188–207. doi: 10.1074/jbc.M116.761213 (PMC5207086; doi:10.1074/jbc.M116.761213)
Supplement: Supplemental Data [file supp_291_50_26188__index.html]

Expansion of Lysine-rich Repeats in Plasmodium Proteins Generates Novel Localization Sequences That Target the Periphery of the Host Erythrocyte — Targeting Role of Repetitive Plasmodium Sequences — Supplemental Data 

# Expansion of Lysine-rich Repeats in *Plasmodium* Proteins Generates Novel Localization Sequences That Target the Periphery of the Host Erythrocyte

## Supplemental Data

**Files in this Data Supplement:**

- Supplemental Data
