# Supplementary material for: Expansion of Lysine-rich Repeats in Plasmodium Proteins Generates Novel Localization Sequences That Target the Periphery of the Host Erythrocyte
Source: J Biol Chem. 2016 Oct 24;291(50):26188–207. doi: 10.1074/jbc.M116.761213 (PMC5207086; doi:10.1074/jbc.M116.761213)
Supplement: Supplemental Data [file supp_M116.761213_Supplementary_MaterialC_M761213.docx]

**
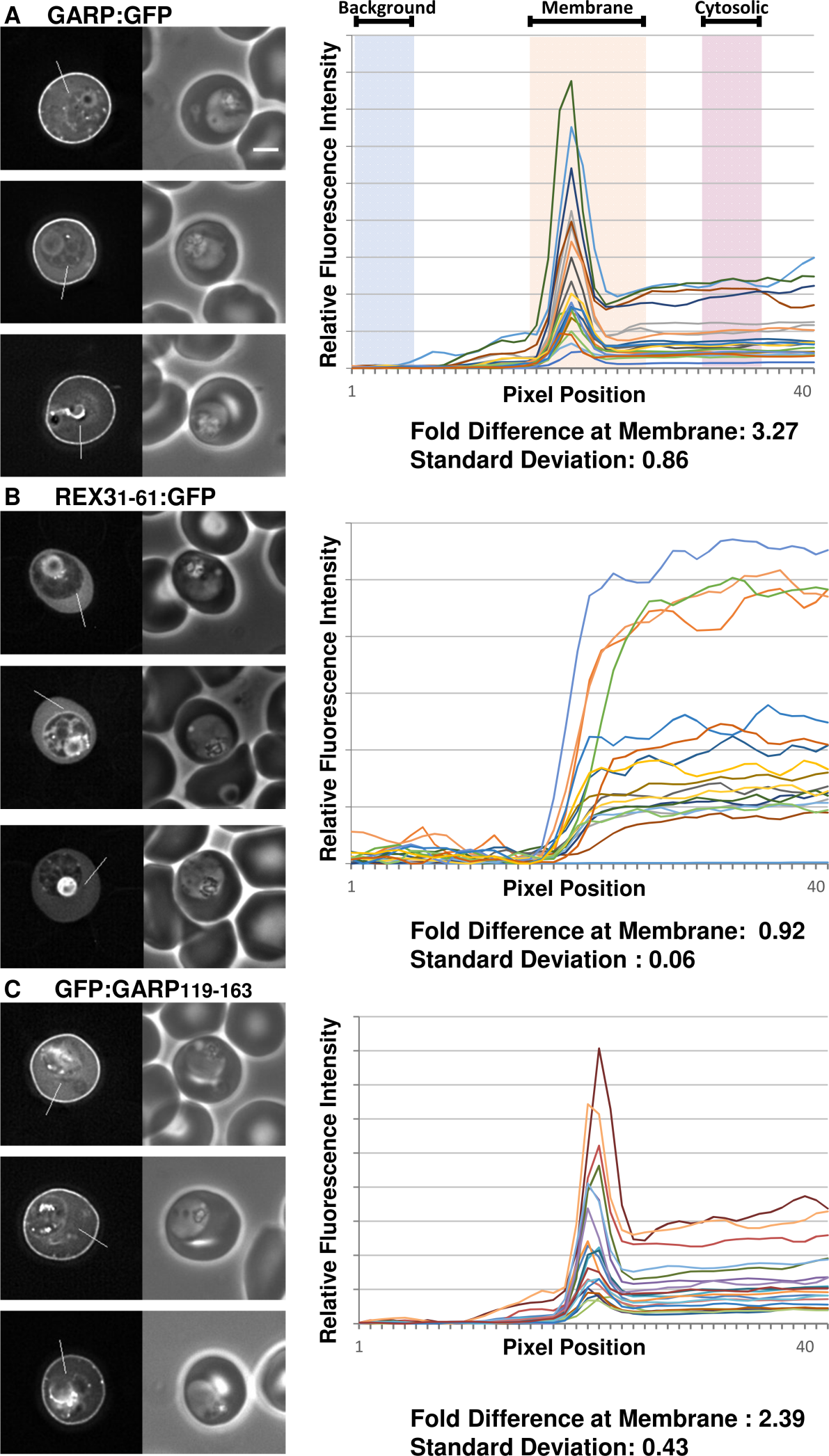
**

**
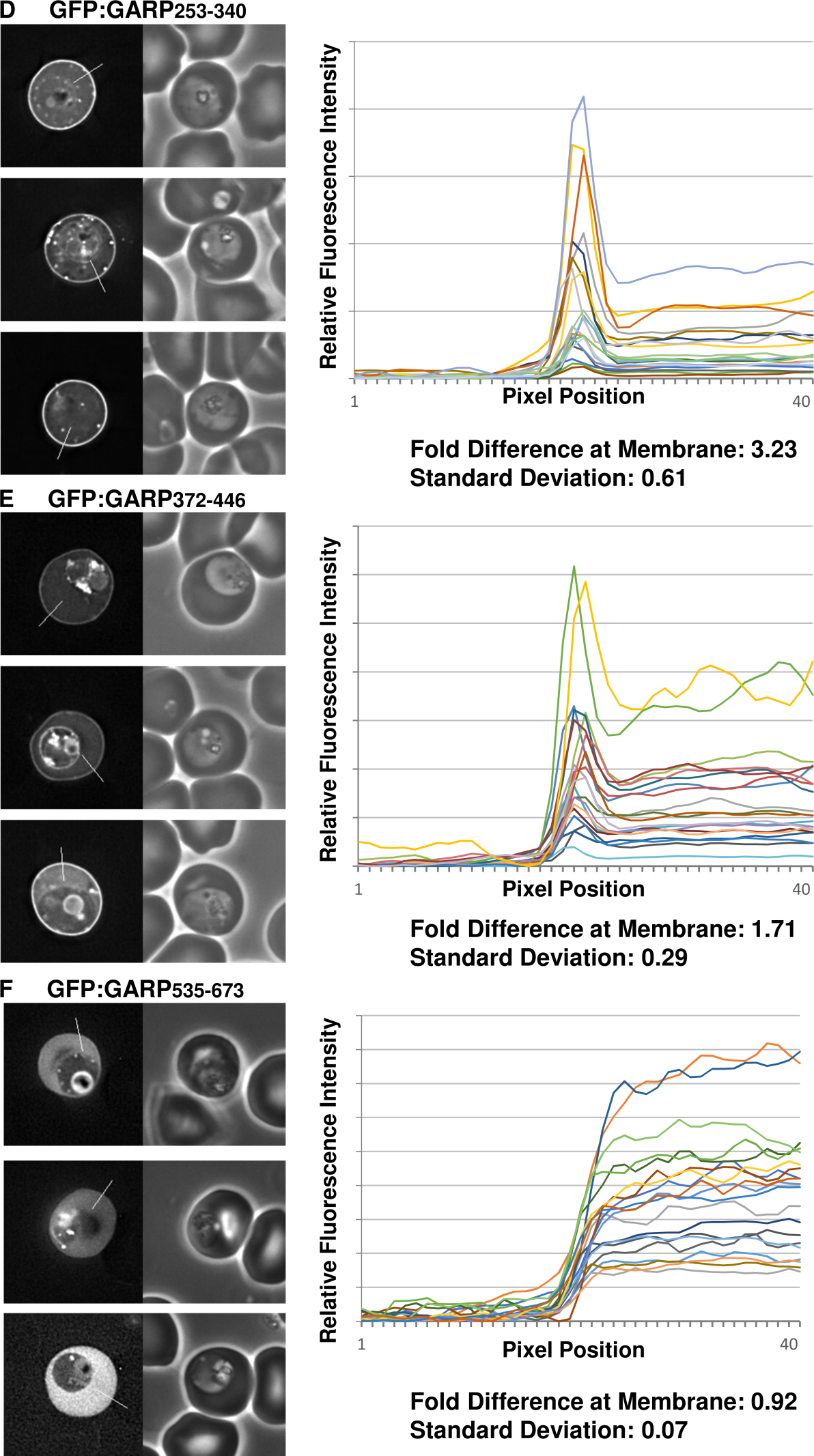

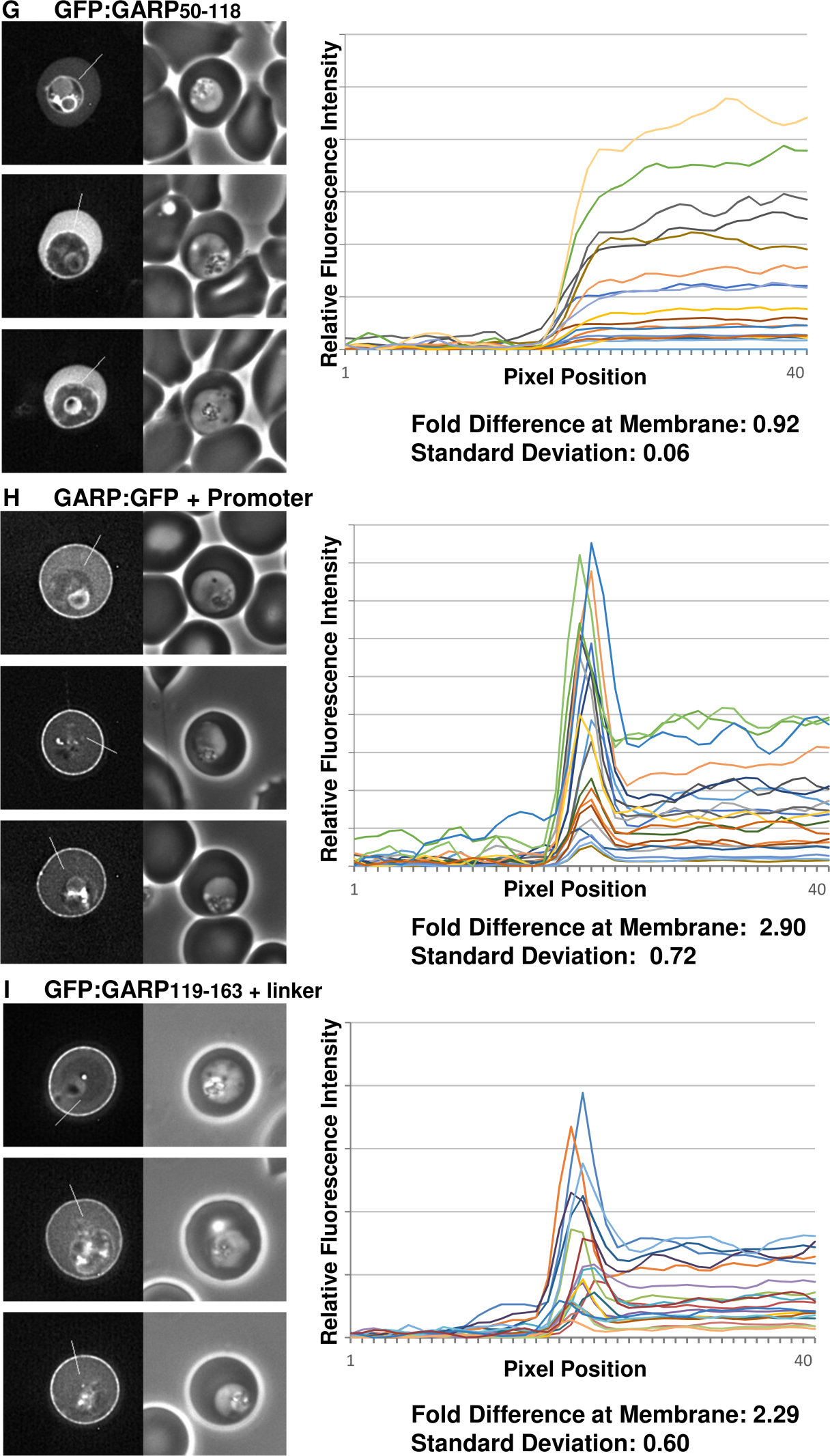

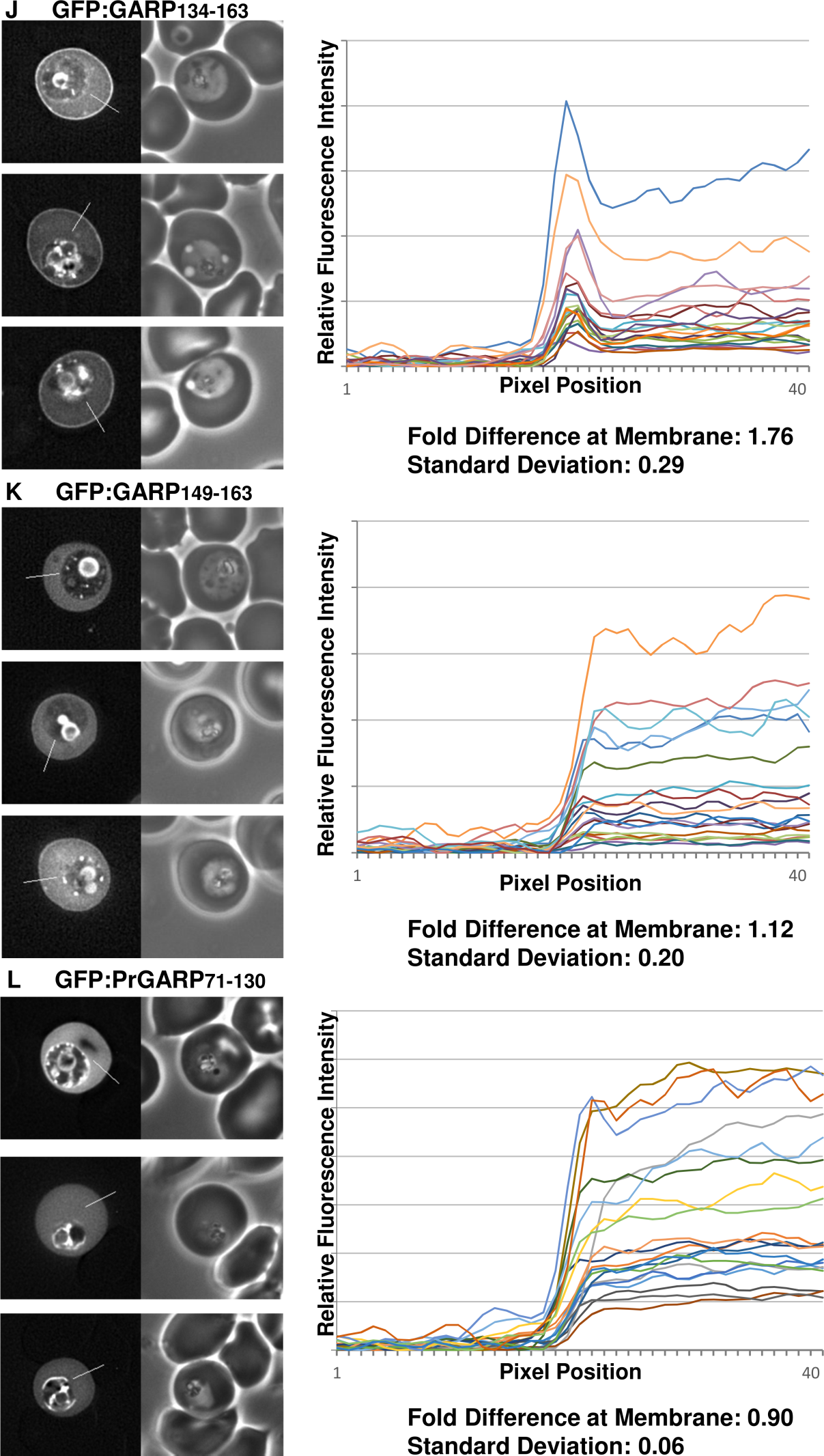

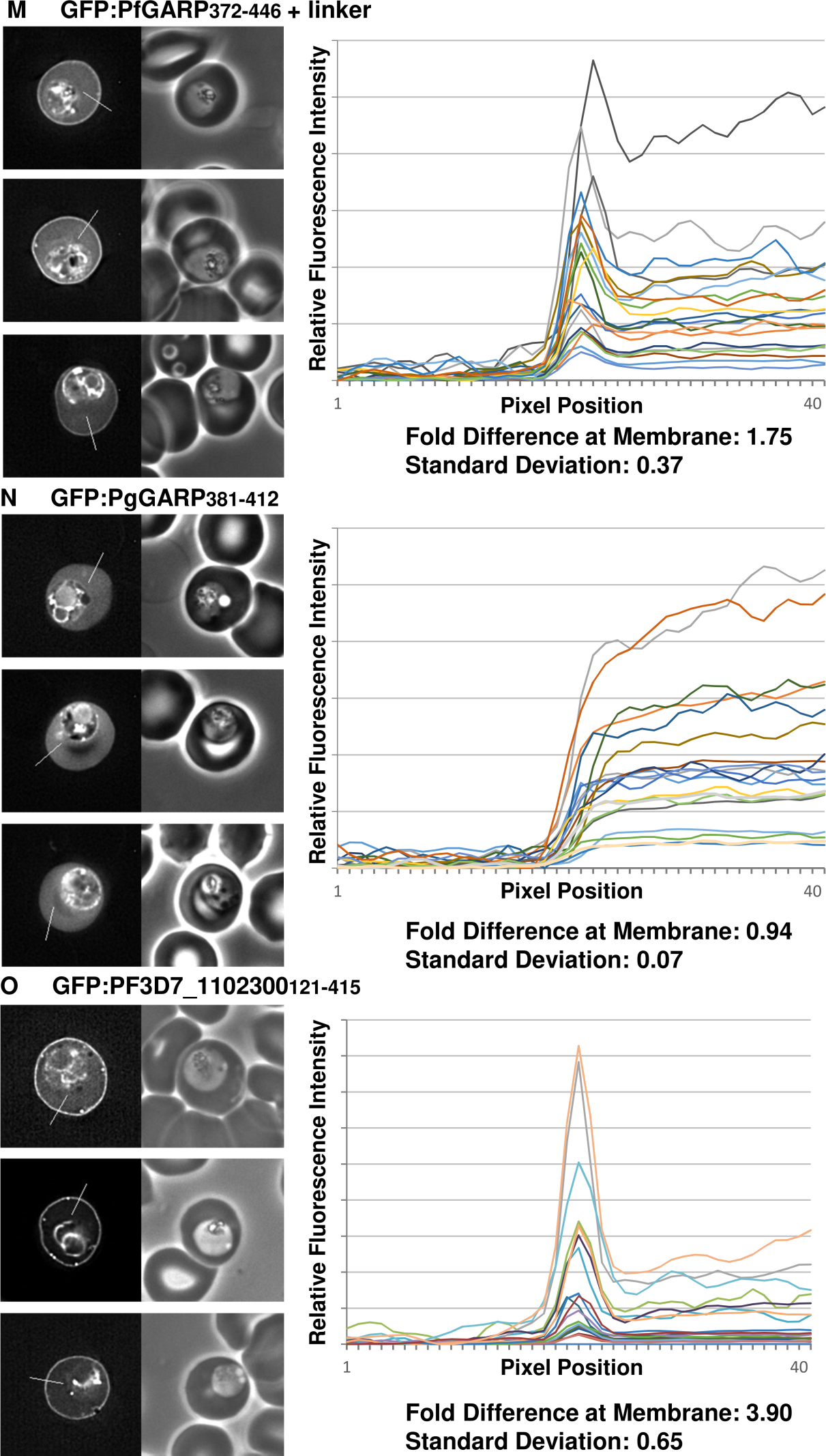

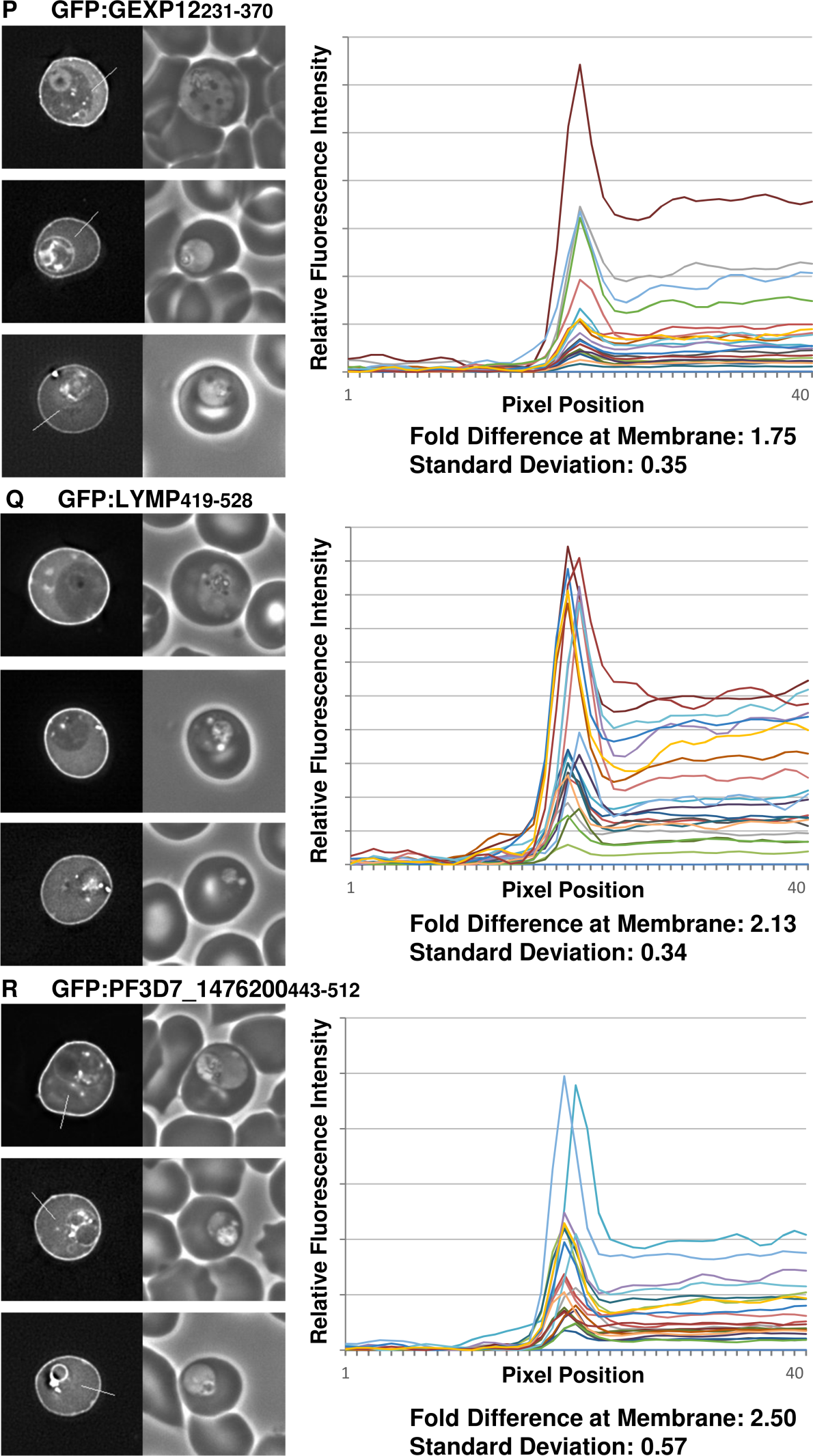

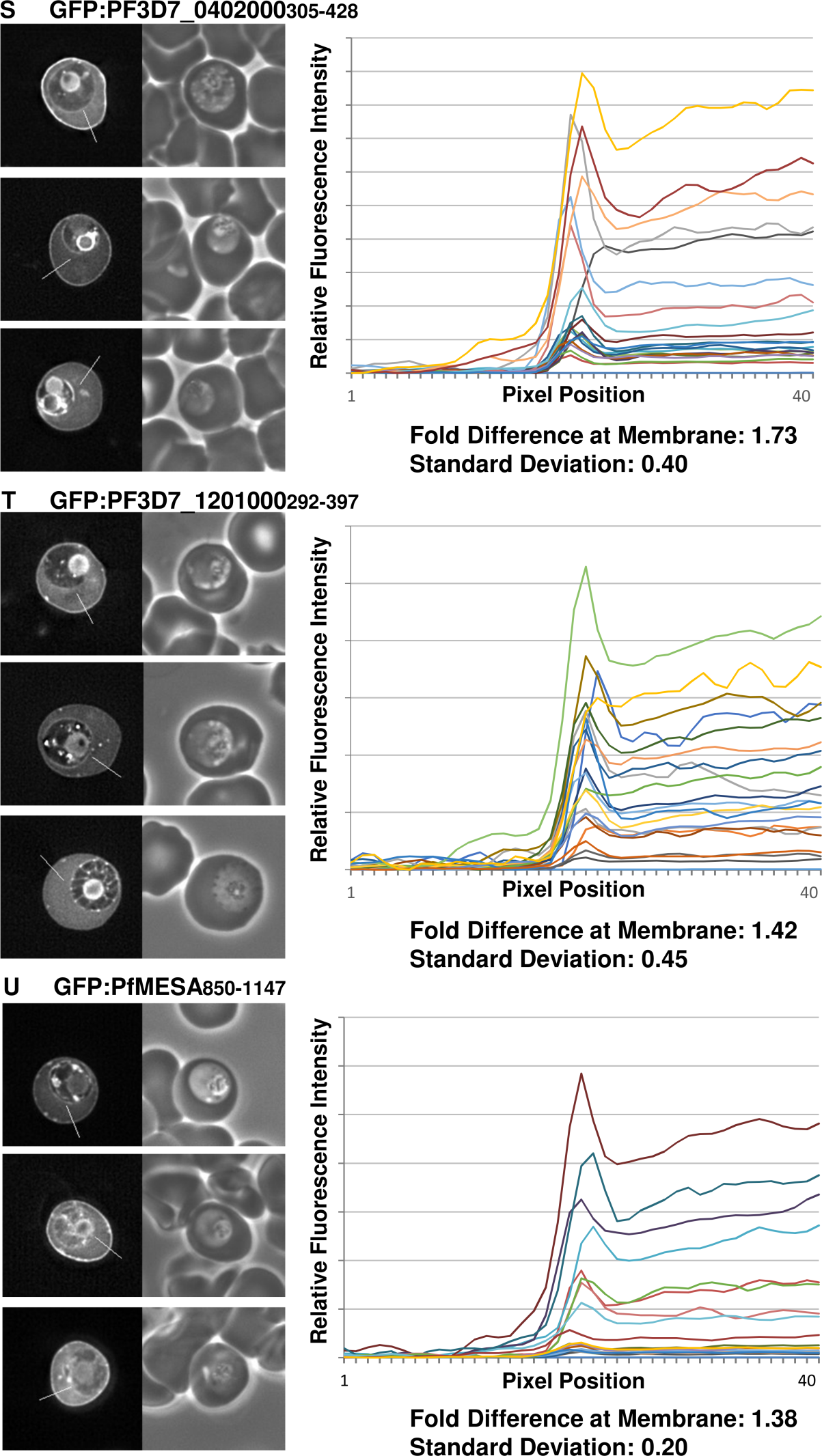

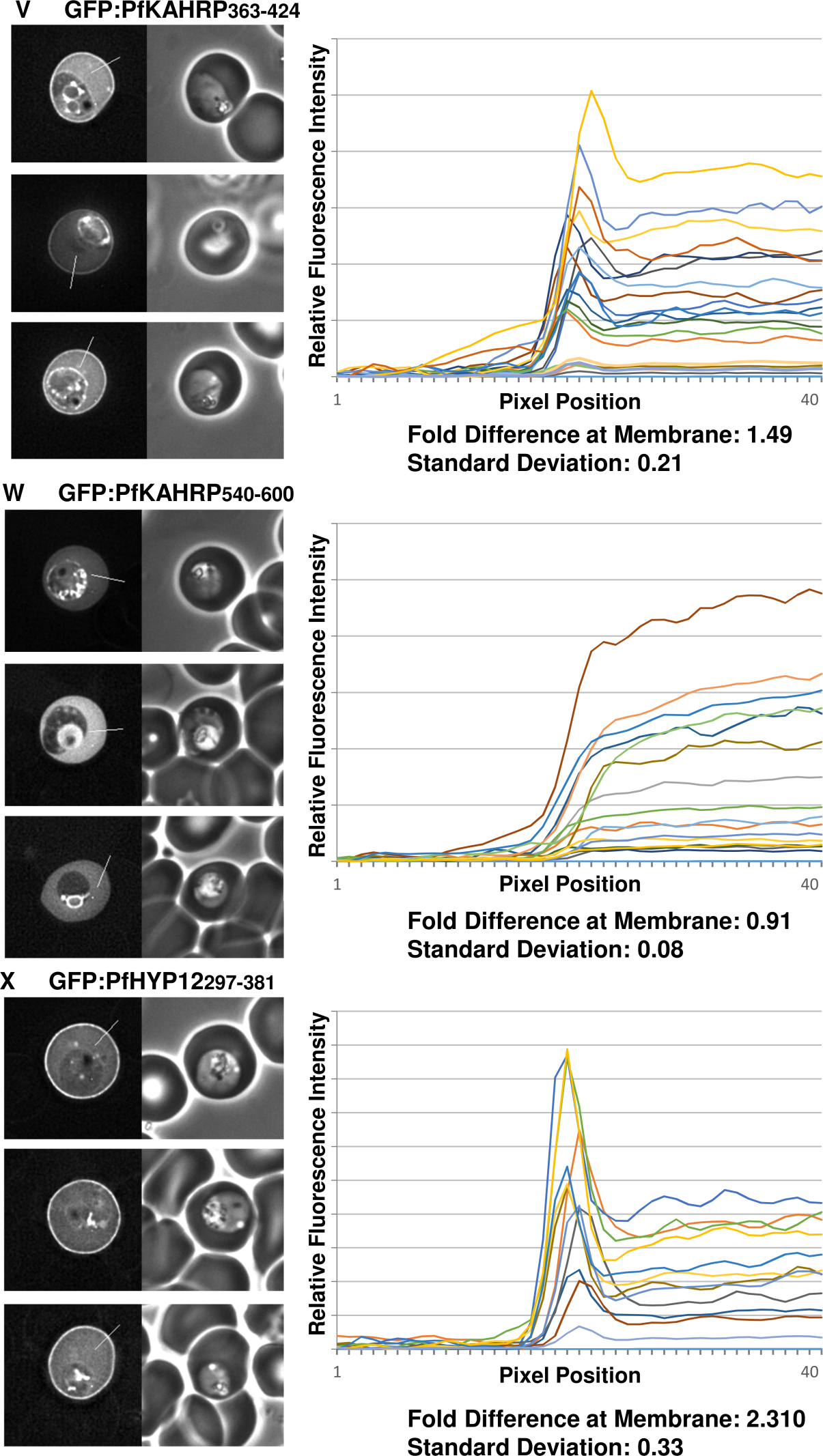

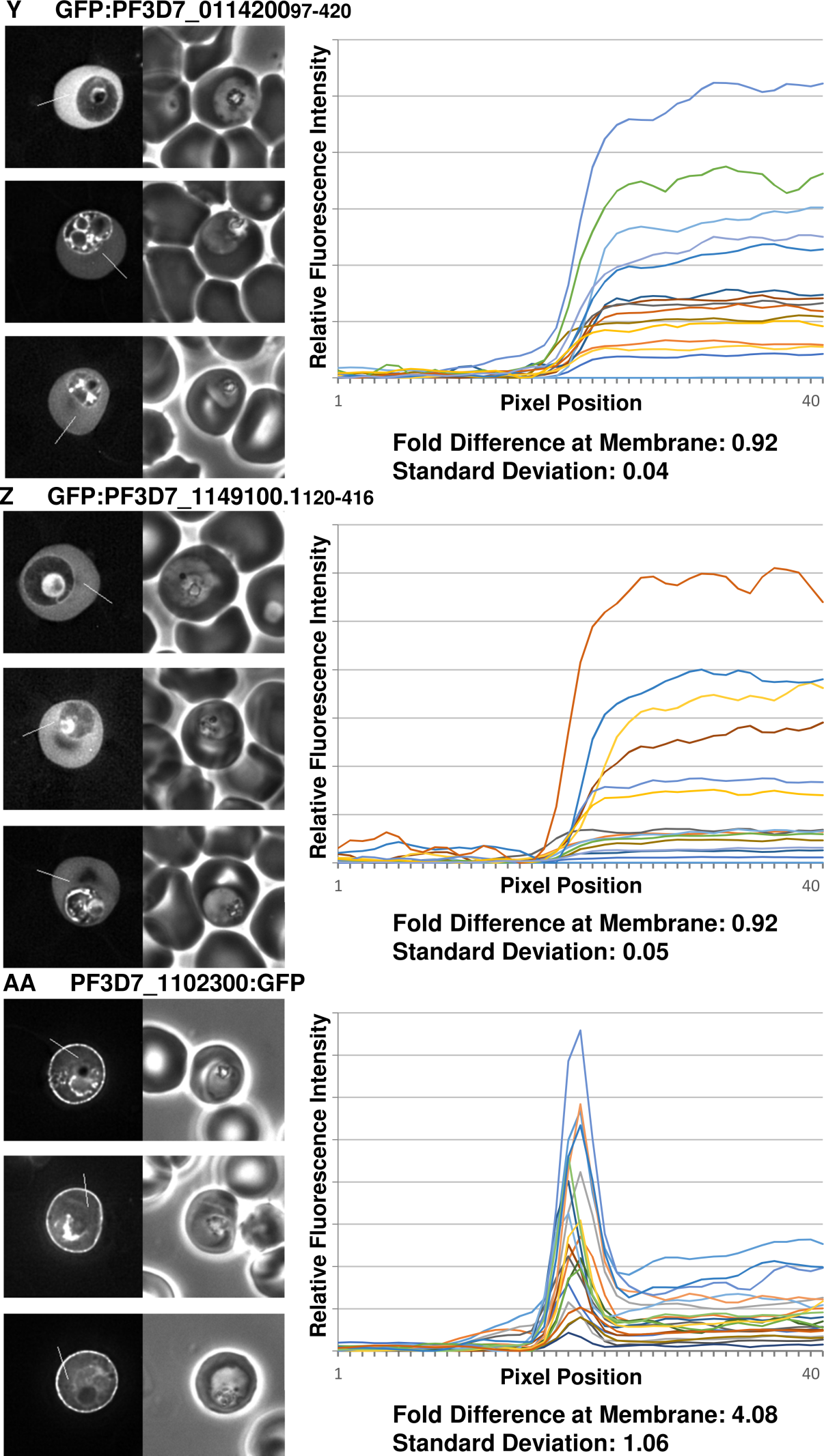

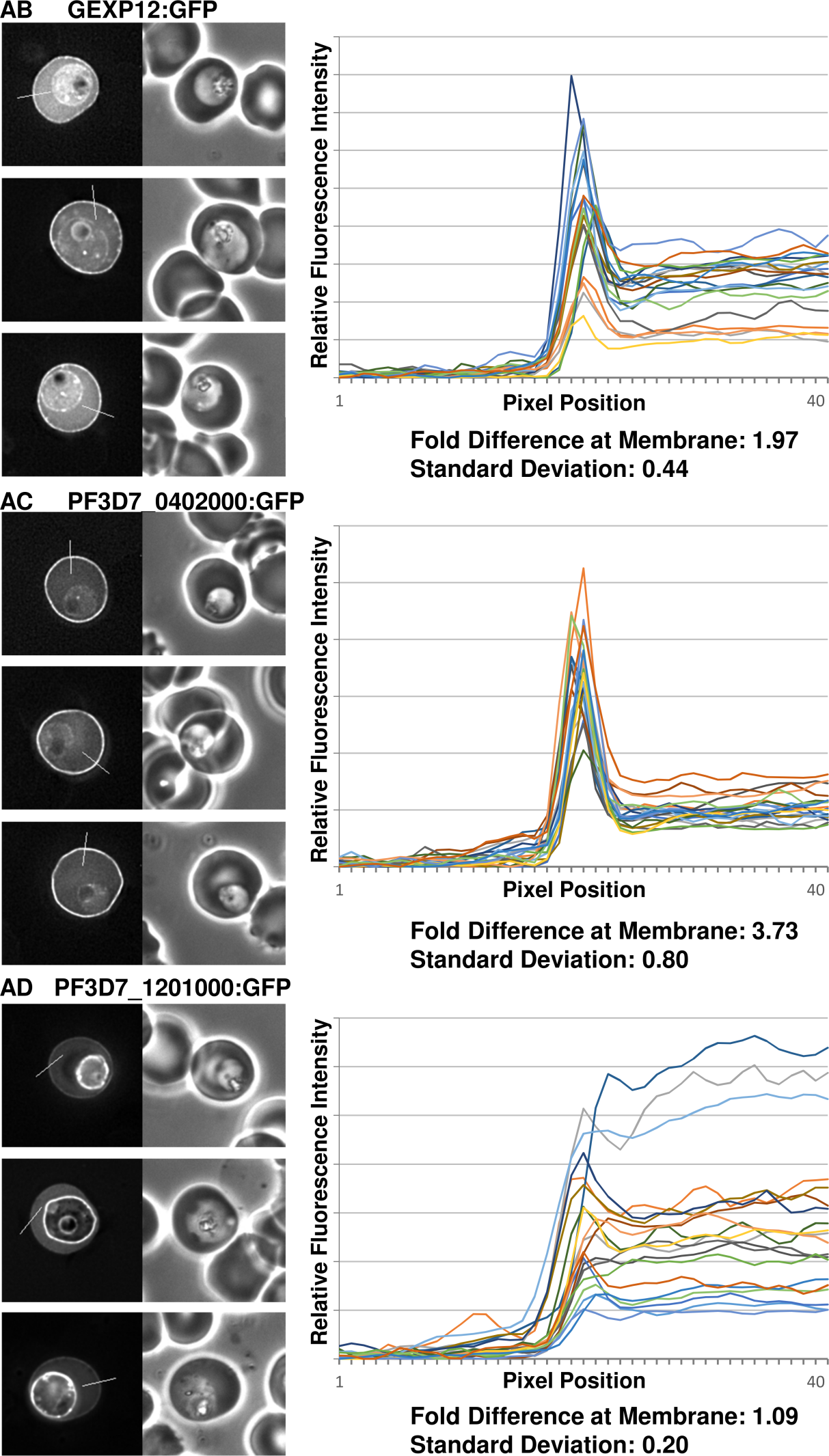

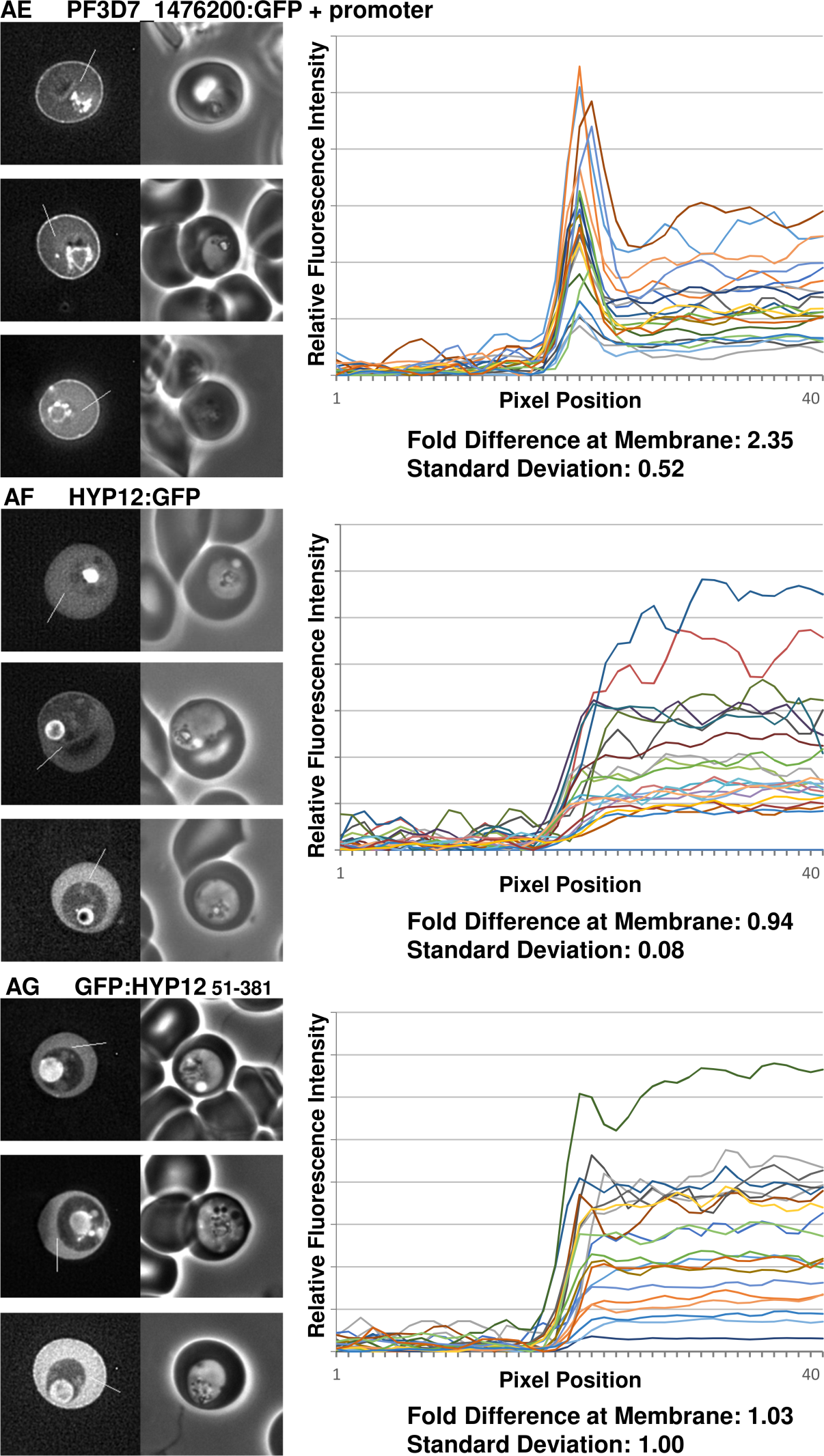

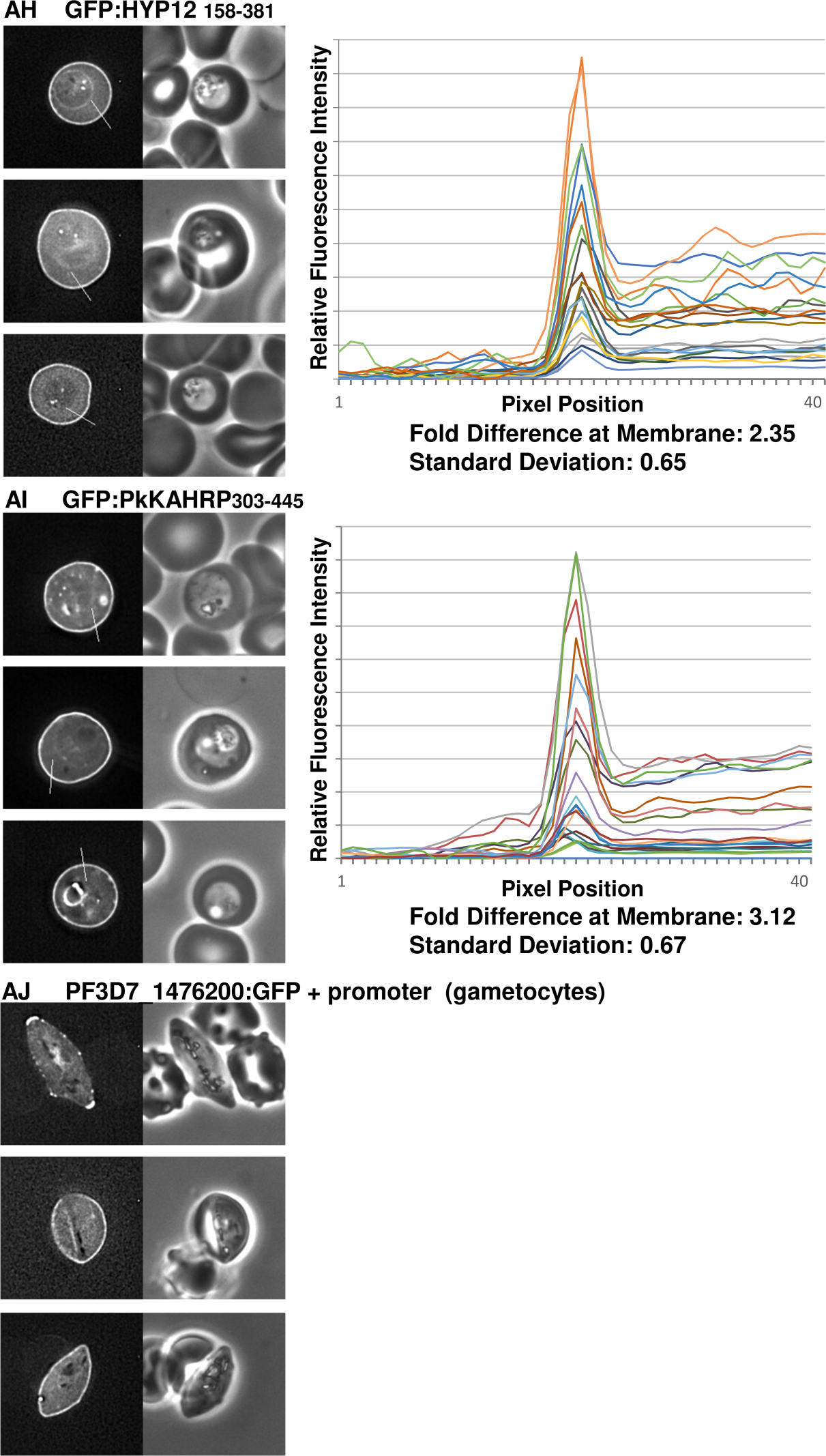
**

**Supplemental Figure 1 - Quantification of GFP fluorescence at the periphery of infected erythrocytes.** (A-AI) Representative GFP fluorescence and phase contrast images are shown on the left and right panels, respectively. ImageJ was used to plot a profile of fluorescence signal intensity across the periphery of the red blood cell as described previously (1). Images were cropped to 170 x 170 pixels and a 40 pixel line was drawn starting outside the cell and ending within the erythrocyte cytoplasm, avoiding the parasite (shown by white lines in fluorescence images). The centre of the line was placed approximately at the erythrocyte membrane. The background signal was taken as the average intensity of the first 5 pixels, while the cytoplasmic signal was calculated as the average intensity of pixels 30-35. The peripheral fluorescence was determined as the maximal signal intensity between pixels 15-25, as indicated. The background signal was subtracted from both cytoplasmic and membrane signals and the difference in fluorescence at the membrane was calculated as the ratio of the normalised membrane to cytosolic signal. This ratio was calculated for 20 infected cells from two separate experiments; the fluorescence intensity profiles of these cells are shown. The average and standard deviation is indicated. (AJ) Additional images of gametocytes at various stages expressing GFP-tagged PF3D7_1102300.

| **Gene ID** | **Alias** | **Consensus Sequence** | **Position Within Protein** | **Repeat Unit Length** | **Number of Repeat Units** | **Error from Consensus** |
| --- | --- | --- | --- | --- | --- | --- |
| PBANKA_0524700 |  | EIYMENKKEEQQQSKKKEKVI | 466-577 | 21 | 5.33 | 0 |
| PBANKA_0711200 |  | EIKKTPTT- | 233-355 | 8 | 15.25 | 0.09 |
| PBANKA_0112600 | fam-b | NDKKTCV | 149-193 | 7 | 6.43 | 0.02 |
| PBANKA_1145400 |  | EEKSEKKSKKKSEKKSEKKSEEKS | 217-295 | 24 | 3.29 | 0.14 |
| PBANKA_0214600 |  | LKNDVANKTQK | 799-839 | 11 | 3.73 | 0.02 |
| PCHAS_0404100 | CSP | PGDK | 79-175 | 4 | 24.25 | 0.11 |
| PCHAS_0521700 |  | KENGEEKVT | 677-714 | 9 | 4.22 | 0.03 |
| PCHAS_0524900 |  | KKKETVIEIYMEDKKGEQQHP | 437-493 | 21 | 2.71 | 0.12 |
| PCHAS_1201300 |  | KEKQEK-ERKE | 957-1043 | 10 | 8.7 | 0.23 |
|  |  | QKPTD | 814-960 | 5 | 29.4 | 0.12 |
| PCHAS_1246800 |  | EYKS | 179-215 | 4 | 9.25 | 0.16 |
| PCHAS_0318300 |  | EEK-VE | 1832-1900 | 5 | 16.60 | 0.01 |
| PCHAS_1370100 |  | DGKKIFEEKKES | 169-201 | 12 | 2.75 | 0.15 |
|  |  | EKKSSNEKKTPG | 199-231 | 12 | 2.75 | 0.06 |
| PCYB_051130 |  | EKKAEKET | 237-278 | 8 | 5.25 | 0.1 |
| PCYB_052240 | RAD | KESKPNV | 375-425 | 7 | 7.29 | 0.2 |
| PCYB_063210 | EBP | EGDKG | 285-313 | 5 | 5.8 | 0.17 |
| PCYB_081160 | TRA | KKSPIIES | 228-279 | 8 | 6.5 | 0.15 |
| PCYB_084720 |  | PKKGAE | 299-361 | 6 | 10.5 | 0.19 |
| PCYB_115490 |  | NEKPKE | 234-287 | 6 | 9 | 0.02 |
|  |  | KGKD | 216-227 | 4 | 3 | 0.15 |
| PKNH_0100400 |  | KEEK | 51-75 | 4 | 6.25 | 0 |
| PKNH_0200400 |  | KEEV | 442-486 | 4 | 11.25 | 0.16 |
| PKNH_0300600 |  | D-KA-KK-EA | 131-284 | 7 | 20 | 0.23 |
| PKNH_0400300 |  | KEEV | 191-288 | 4 | 24.5 | 0.07 |
| PKNH_0623000 |  | GKKECPFKAQNSESDKCA | 462-703 | 18 | 13.44 | 0.04 |
| PKNH_0807900 |  | KKEE | 69-110 | 4 | 10.5 | 0.1 |
| PKNH_0841300 |  | EKGAQKPGQKKVQEKKDSN | 276-320 | 19 | 2.37 | 0 |
|  |  | KKEE | 69-170 | 4 | 25.5 | 0.06 |
| PKNH_0900500 |  | KYENG | 162-361 | 5 | 40 | 0.17 |
| PKNH_1100500 |  | KYE-NG | 162-263 | 5 | 20.2 | 0.17 |
| PKNH_1149200 |  | KEK-E-QEKK- | 209-270 | 8 | 7.25 | 0.21 |
| PKNH_1149700 |  | KKEE | 69-142 | 4 | 18.5 | 0.11 |
| PKNH_1246800 |  | GNKYENKHEEKL | 355-379 | 12 | 2.08 | 0.16 |
|  |  | YNDK | 322-350 | 4 | 7.25 | 0.03 |
| PKNH_1247400 |  | GAQKPAQQKVQEKKDSNEK | 344-491 | 19 | 7.79 | 0.03 |
|  |  | EEKK | 77-232 | 4 | 39 | 0.1 |
| PKNH_1304600 |  | PPKGTKKKTPTEETEQQA | 153-188 | 18 | 2 | 0.03 |
| PKNH_1313400 |  | PTPKKE | 266-299 | 6 | 5.67 | 0.09 |
| PKNH_1325700 | KAHRP | PTVSQPPK | 304-363 | 8 | 7.5 | 0.08 |
|  |  | EQAKK | 364-432 | 5 | 13.8 | 0 |
| PKNH_1325800 |  | ETEKQDKPKYTYGSYKYPTVK | 313-404 | 21 | 4.38 | 0.12 |
|  |  | KKEKEKKDKKE | 917-959 | 11 | 3.91 | 0.2 |
| PKNH_1441900 |  | KKKEKEKEKE | 242-271 | 10 | 3 | 0.1 |
| PKNH_1473200 |  | KEEK | 51-111 | 4 | 15.25 | 0 |
| PRCDC_0053100 | RIFIN | KRQKHKEQRDKNIQKIIEKDKR | 82-125 | 22 | 2 | 0 |
| PRCDC_0060600 | PHISTB | KENNDNE | 256-269 | 7 | 2 | 0.14 |
| PRCDC_0111200 | GARP | KKERKQKEKEMKKQEKIEKK-- | 229-296 | 20 | 3.4 | 0.19 |
| PRCDC_0112400 | EPF3 | DHMK | 105-212 | 4 | 27 | 0.14 |
| PRCDC_0201000 | EMP3 | GLKENAELKNKELRNKGSD | 694-793 | 19 | 5.26 | 0.03 |
|  |  | KNKDI | 796-818 | 5 | 4.6 | 0.26 |
| PRCDC_0201100 | KAHRP | GE-KKKSKKNKD-NDDAESFKSKKSVKEK | 362-452 | 27 | 3.3 | 0.08 |
|  |  | KGATKEASTS | 545-613 | 10 | 6.9 | 0.09 |
| PRCDC_0500100 | MESA | EKND-EKKDKVLG-EGDKEDVK | 402-473 | 20 | 3.5 | 0.17 |
| PRCDC_0500500 | PIESP2 | KHKEDH | 184-232 | 6 | 8.17 | 0.06 |
| PRCDC_0506400 | SUB3 | KNNDS | 246-294 | 5 | 9.8 | 0.14 |
| PRCDC_0531400 | LYMP | ENKKAGS | 437-494 | 7 | 8.29 | 0.09 |
| PRCDC_0723000 | FIKK 7.1 | KKEDKSCMKKTHGNKAEDE | 226-305 | 19 | 4.21 | 0.08 |
|  |  | DLIKNKEG | 84-176 | 8 | 11.62 | 0.14 |
| PRCDC_0727700 | PTP4 | FVDNKEKTLGKHE-HHEEHVKGK | 1210-1444 | 22 | 10.68 | 0.2 |
| PRCDC_1001500 | PTP5 | NETEKKTDQ | 224-262 | 9 | 4.33 | 0.05 |
| PRCDC_1037500 | GSP | EKEEKIKKKKVIEKKK | 1513-1547 | 16 | 2.19 | 0.28 |
|  |  | E-PK-KEK--AP | 1623-1776 | 8 | 18.75 | 0.21 |
|  |  | KDVKAKHK | 1550-1566 | 8 | 2.12 | 0.12 |
|  |  | EEKFLK | 370-381 | 6 | 2 | 0.17 |
|  |  | D-EK | 331-366 | 3 | 11.67 | 0.11 |
| PRCDC_1100800 |  | ERKEREEREKQ | 134-392 | 11 | 23.55 | 0.26 |
| PRCDC_1146600 | PHISTc | KECIPKECIK | 263-332 | 10 | 7 | 0.2 |
| PRCDC_1249000 | LRR | DKKEDVDNEKYG | 529-593 | 12 | 5.42 | 0.18 |
| PRCDC_1400500 |  | QKKKKPSKYDDIRRFGEPT | 73-139 | 19 | 3.53 | 0.13 |
| PRCDC_1475300 | PHISTB | KKEEDV | 372-404 | 6 | 5.5 | 0.09 |
| PRCDC_1475600 |  | NKEENKDN | 471-505 | 8 | 4.38 | 0.06 |
| PVX_002507 | Pv-fam-b | GAMKNDTKKTPAKR | 85-282 | 14 | 14.14 | 0.08 |
| PVX_002535 | PHIST | LEEKLNVKKLQELVKLKD | 87-143 | 18 | 3.17 | 0.19 |
| PVX_003535 |  | NEMGK | 192-247 | 5 | 11.2 | 0.11 |
| PVX_081440 |  | KKRLKEEE | 121-142 | 8 | 2.75 | 0.18 |
|  |  | RKERK | 92-103 | 5 | 2.4 | 0.17 |
| PVX_081835 | KAHRP | KKETK | 526-564 | 5 | 7.8 | 0.08 |
|  |  | EINTE | 563-642 | 5 | 16 | 0.14 |
|  |  | EKKK- | 686-713 | 4 | 6.75 | 0.07 |
| PVX_086900 |  | RSHKKD | 713-784 | 6 | 12 | 0.18 |
| PVX_089435 | RAD | KKPTA-QV | 436-483 | 7 | 6.86 | 0.12 |
|  |  | EKKPDGK | 484-504 | 7 | 3 | 0.19 |
|  |  | GKPVE | 503-515 | 5 | 2.6 | 0.08 |
| PVX_089790 | RAD | KGKTPD | 238-370 | 6 | 22.17 | 0.09 |
| PVX_089795 | RAD | KGEAK | 264-318 | 5 | 11 | 0.07 |
| PVX_089810 | RAD | TKPKAG | 238-265 | 6 | 4.67 | 0.04 |
| PVX_097575 | TRA | PQSKAKQQ | 962-1054 | 8 | 11.62 | 0.14 |
| PVX_110825 | Pv-fam-D | KNDDKDSFISGKS | 1010-1061 | 13 | 4 | 0.13 |
| PVX_110835 |  | EGDQ--D-GK-EDKGEEDEDGK | 258-297 | 18 | 2 | 0.22 |
|  |  | CPYKDQSVDKKE | 772-823 | 12 | 4.33 | 0.1 |
|  |  | KKTANVKKGAEP | 1200-1226 | 12 | 2.25 | 0.04 |
|  |  | DK-D-KDDK | 293-337 | 7 | 6 | 0.32 |
|  |  | EEEAKKL | 1146-1195 | 7 | 7.14 | 0.16 |
| PVX_118682 | EMP3 | EAKKPEVKKT | 1001-1030 | 10 | 3 | 0.17 |
| PVX_119225 |  | KKAAAP | 307-362 | 6 | 9.33 | 0.14 |
| PY17X_0114200 | fam-b | KKADVND | 284-377 | 7 | 13.43 | 0.11 |
| PY17X_0114400 | fam-b | DNKLDDK | 175-198 | 7 | 3.43 | 0 |
| PY17X_0216300 |  | KTEKIKNEVSN | 603-688 | 11 | 7.82 | 0.13 |
| PY17X_0405400 | CSP | KDDLPKEEK | 89-122 | 9 | 3.78 | 0.12 |
| PY17X_0526100 |  | EKVIEIYMEDKKGKEQESKKK | 462-766 | 21 | 14.52 | 0.25 |
| PY17X_0711400 |  | EIKKAPTSTEIKKASTST | 233-307 | 18 | 4.17 | 0.13 |
| PY17X_0932500 | Tyr-tRNA ligase | EELKN | 391-445 | 5 | 11 | 0.07 |
| PY17X_1112100 |  | EIDKSIKKEEEHIKK- | 120-173 | 15 | 3.6 | 0.26 |
| PY17X_1203700 |  | QVTDK | 1242-1313 | 5 | 14.4 | 0.15 |
|  |  | QVSDK | 677-768 | 5 | 18.4 | 0.14 |
|  |  | QVTDK | 787-1070 | 5 | 56.8 | 0.12 |
| PY17X_1440700 | GyrA | KDE | 125-164 | 3 | 13.33 | 0.15 |

**Supplemental Table 1 – Proteins from multiple parasite species contain lysine-rich repeat sequences predicted to target to the erythrocyte periphery.** Gene identifiers as follows: *P. berghei* – PBANKA, *P. chabaudi* – PCHAS, *P. cynomolgi* – PCYB, *P. knowlesi* – PKNH, *P. reichenowi* – PRCDC, *P. vivax* – PVX and *P. yoelli* – PY17X. The consensus sequence, position within the protein, repeat unit length, number of repeat units, and the error from consensus were defined by XSTREAM (2).

| **Table 2A** | | | |
| --- | --- | --- | --- |
| **Gene Name** | **Location of Potential Error** | **Nature of Potential Error** | |
| PFCD01_GARP | 360 | Frame shift (Insertion) | |
|  | 766 | Frame shift (Insertion) | |
| PFGN01_GARP | 781 | Frame shift (Insertion) | |
| PFML01_KAHRP | 1020 | Frame shift (Insertion) | |
| PFML01_MESA | 3030 | Frame shift (Insertion) | |
| PFCD01_MESA | 1190 | Frame shift (Insertion) | |
| PFGN01_PF3D7_1102300 | 978 | Frame shift (Insertion) | |
| PFSD01_PF3D7_1102300 | 1013 | Frame shift (Insertion) | |
| PFGA01_PF3D7_0402000 | 1082 | Frame shift (Deletion) | |
| PFML01_PF3D7_1201000 | 995 | Frame shift (Insertion) | |
| PFTG01_PF3D7_1201000 | 995 | Frame shift (Insertion) | |
| **Table 2B**  **Location of Potential Error**  **Nature of Potential Error** | | | |
| PI C922_04319 | | 110-295 | Un-annotated intron |
|  |  | 365 | Point mutation (Stop codon) |
|  |  | 749 | Point mutation (Stop codon) |
|  |  | 1244 | Point mutation (Stop codon) |
| PI C922_02878 | | 112-243 | Un-annotated intron |
|  |  | 74 | Frame shift (Deletion) |
|  |  | 1003 | Point mutation (Stop codon) |
|  |  | 1672 | Point mutation (Stop codon) |
| PFR AK88_04565 | | 112-256 | Un-annotated intron |
|  |  | 996 | Assembly gap (skipped) |
|  |  | 1625 | Assembly gap (skipped) |
|  |  | 2737 | Assembly gap (incomplete sequence) |
| PCYB_001100 | | 109-284 | Un-annotated intron |
|  |  | 3 | Point mutation in start codon |
|  |  | 109-284 | Un-annotated intron |
|  |  | 639 | Point mutation (Stop codon) |
|  |  | 1217 | Assembly gap (incomplete sequence) |
| PCYB_127900 | | 97-246 | Un-annotated intron |
|  |  | 46 | Frame shift (deletion) |
| PCYB_042840 | | 112-242 | Un-annotated intron |
|  |  | 37 | Frame shift (deletion) |
|  |  | 1080 | Frame shift (deletion) |
|  |  | 1716-2157 | Reverse complement |
|  |  | 2129 | Frame shift (insertion) |

**Supplemental Table 2 – Annotation of introns and location of potential frameshift mutations. (**A) Sequences containing frameshift mutations in PACBIO sequences were restored. Where multiple sequences were found (for PfML01_KAHRP, PfML01_MESA, PfTG01_MESA and PfTG01_1476200), the sequence containing the fewest frameshift mutations was used (either one or zero mutations). (B) EKAL-domain proteins modified from their deposited protein sequences. Introns were annotated manually for all sequences indicated. For protein PFR AK88_04565, apparent assembly gaps were present in the protein sequence, which were skipped in accordance to the protein annotation within the European Nucleotide Archive (ENA). Assembly gaps within PFR AK 88_04565 and PCYB_001100 result in a truncated sequence with no stop codon. A section of gene PCYB_042840 appears to be reverse complemented within the assembled sequence, which was modified in-frame with flanking regions. All mutations may be caused by sequencing errors or may be true mutations.

**Supplemental Material References**

1. Tarr, S. J., Moon, R. W., Hardege, I., and Osborne, A. R. (2014) A conserved domain targets exported PHISTb family proteins to the periphery of Plasmodium infected erythrocytes. *Molecular and Biochemical Parasitology* **196**, 29-40

2. Newman, A. M., and Cooper, J. B. (2007) XSTREAM: A practical algorithm for identification and architecture modeling of tandem repeats in protein sequences. *Bmc Bioinformatics* **8**, 19
